# Large Spatial Scale Variability in Bathyal Macrobenthos Abundance, Biomass, α- and β-Diversity along the Mediterranean Continental Margin

**DOI:** 10.1371/journal.pone.0107261

**Published:** 2014-09-16

**Authors:** Elisa Baldrighi, Marc Lavaleye, Stefano Aliani, Alessandra Conversi, Elena Manini

**Affiliations:** 1 Institute of Marine Sciences, National Research Council (ISMAR-CNR), Ancona, Italy; 2 Department of Marine Ecology, Royal Netherlands Institute for Sea Research (NIOZ), Texel, The Netherlands; 3 Institute of Marine Sciences, National Research Council (ISMAR-CNR), La Spezia, Italy; 4 Marine Institute, Plymouth University, Plymouth, United Kingdom; Technical University of Denmark, Denmark

## Abstract

The large-scale deep-sea biodiversity distribution of the benthic fauna was explored in the Mediterranean Sea, which can be seen as a miniature model of the oceans of the world. Within the framework of the BIOFUN project (“Biodiversity and Ecosystem Functioning in Contrasting Southern European Deep-sea Environments: from viruses to megafauna”), we investigated the large spatial scale variability (over >1,000 km) of the bathyal macrofauna communities that inhabit the Mediterranean basin, and their relationships with the environmental variables. The macrofauna abundance, biomass, community structure and functional diversity were analysed and the α-diversity and β-diversity were estimated across six selected slope areas at different longitudes and along three main depths. The macrobenthic standing stock and α-diversity were lower in the deep-sea sediments of the eastern Mediterranean basin, compared to the western and central basins. The macrofaunal standing stock and diversity decreased significantly from the upper bathyal to the lower bathyal slope stations. The major changes in the community composition of the higher taxa and in the trophic (functional) structure occurred at different longitudes, rather than at increasing water depth. For the β-diversity, very high dissimilarities emerged at all levels: (i) between basins; (ii) between slopes within the same basin; and (iii) between stations at different depths; this therefore demonstrates the high macrofaunal diversity of the Mediterranean basins at large spatial scales. Overall, the food sources (i.e., quantity and quality) that characterised the west, central and eastern Mediterranean basins, as well as sediment grain size, appear to influence the macrobenthic standing stock and the biodiversity along the different slope areas.

## Introduction

Different studies have been conducted worldwide to define latitudinal and longitudinal diversity patterns of marine biodiversity [1]–[3], which have often been coupled to the bathymetric trends of organisms [4]–[7]. Nevertheless, these patterns and the mechanisms involved in their generation are still far from being understood [8]–[11].

Rex and co-authors [12] presented the first global-scale analysis of the bathymetric patterns of the standing stock (i.e., abundance, biomass) for four major size classes of deep-sea biota: prokaryotes, metazoan meiofauna, macrofauna and megafauna. For the last three of these benthic components, they reported that the community standing-stock decreases with depth, and interpreted this to be a universal phenomenon. This is, however, controversial, and should be related to the taxon considered each time within each benthic size component [13]–[15]. Similarly, for the bathymetric trends in the standing stock, the well-known ‘hump-shape’ distribution in species richness with a diversity maximum at mid-slope depths might not always be the rule [16]–[18].

The spatial heterogeneity of benthic communities is usually related to the different environmental conditions encountered [19], although our understanding of the mechanisms that might act as drivers for the benthic fauna distribution and diversity in the deep sea is still limited [20]. Nevertheless, some factors are usually invoked, including: substrate heterogeneity [21], [22]; water circulation [4], [23]; oxygen availability [19]; productivity and microbial activity [24]; and food resources [25], [26]. Food availability in particular, which is mainly determined by the surface-water primary production [27], and which can decrease sharply with depth, appears to be a major factor that influences the standing stock and the diversity of the deep-benthic communities that depend on this allochthonous organic-matter input [10], [28], [29]. Degradation processes in the water column that affect the quantity and quality of the organic matter that reaches the bottom have also been suggested to have an influence on benthic communities [30].

In the Mediterranean Sea, an overall decrease in benthic abundance, biomass and species richness has been observed from northwestern to southeastern areas for the meiofauna [29], [31], macrofauna [4], [32]–[34] and megafauna [35], [36]. According to different studies [37], [38], the west-east gradient of decreasing surface-water productivity of the Meditterranean Sea is reflected in an increasing paucity of the food that reaches the sea floor moving eastwards. Such a gradient might thus be responsible for the decrease in deep benthic fauna abundance and biomass from west to east. Danovaro and co-authors [39] have shown that the effects of the food supply, and consequently the derived longitudinal trend in the Mediterranean, might be inconsistent across different components of the benthic diversity.

There have been few recent quantitative studies that have dealt with the large-scale patterns of distribution and diversity of the deep Meditteranean macrofauna and/or have addressed the influence of environmental conditions on these macrofauna communities. After a qualitative review by Fredj and Laubier [40] and some descriptive studies that were conducted on bathyal and abyssal macrofaunal organisms or focused on specific taxonomic groups [34], [41]–[54], the most recent studies have reported that the bathyal macrofauna communities in the eastern basin are characterised by low abundance and low diversity, with respect to the western basin; these decrease sharply with depth and are strongly related to food availability [32], [55], [56]. The scant information regarding the macrofauna of the western basin comes from a limited number of slope and canyon areas [23], [26], [57], [58] on the northwestern side of this basin, and shows a decrease in both biomass and density with depth.

We hypothesised that the macrofauna standing stock and diversity change with longitude and depth and according to the major influential environmental variables that characterise the systems investigated.

The main aims were thus to study the deep Mediterranean, in order to:

- assess the longitudinal-related (over >1000 km, and from 3° E to 25° E) and depth-related (1200 m to 2800 m water depth) trends in the macrofaunal abundance, biomass and diversity (i.e., structural and functional diversity), and the influence of the environmental variables on the macrobenthic populations;

-investigate and quantify the macrofaunal β-diversity [60] between: (i) the three basin areas; (ii) the slopes within the same basin; and (iii) stations at different depths.

## Materials and Methods

### Ethics statement

All of the field activities were approved by the local national authorities. The sampling areas were not privately owned or protected in any way, and no endangered or protected species were involved in this study.

### Sampling plan

**Figure 1:**
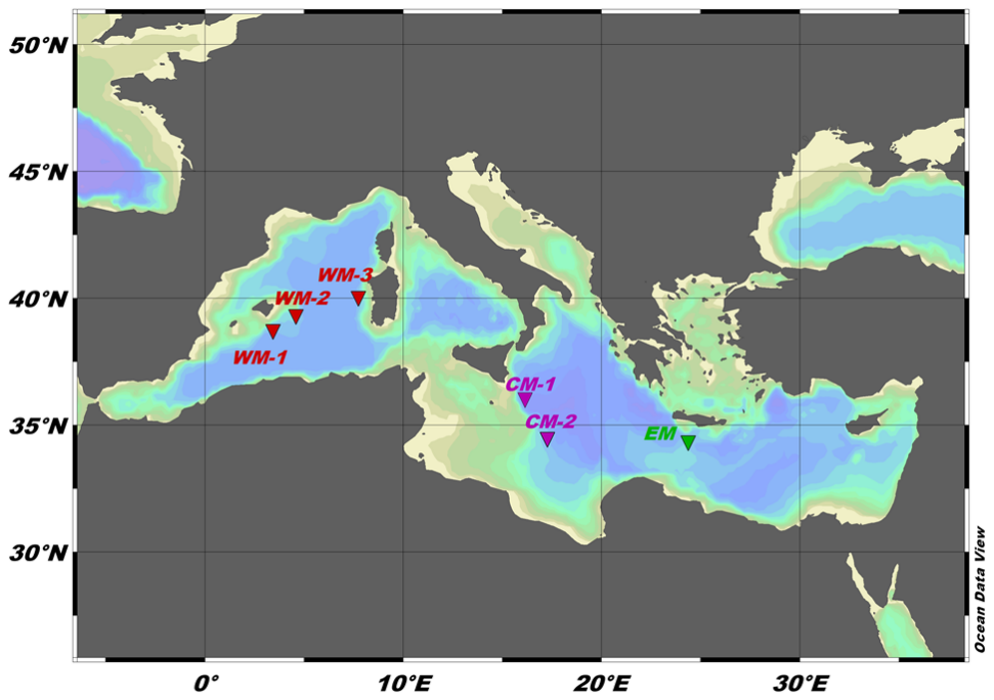
Map of the study area and sampling sites. Red triangles, western Mediterranean basin (WM-1, -2, -3); purple triangles, central Mediterranean basin (CM-1, -2); green triangle, eastern Mediterranean basin (EM). WM-1, Balearic slope 1; WM-2, Balearic slope 2; WM-3, Sardinia slope; CM-1, Maltese slope; CM-2, Ionian slope; EM, Cretan slope.

To achieve our aims, sediment samples were collected from the deep Mediterranean Sea, covering a large spatial scale (over >1000 km) of investigation across different depths (from 1200 m to 2800 m) and longitudes (from 3° E to 25° E). Six cruises (see Table S1 for details) were performed in the Mediterranean Sea on board the R/V Urania (2008–2010), R/V Pelagia (2009) and R/V Meteor (2010), within the framework of the BIOFUN project (“Biodiversity and Ecosystem Functioning in Contrasting Southern European Deep-sea Environments: from viruses to megafauna”), to collect biological and environmental samples from different continental slope systems. The relationship(s) between the macrofauna standing stock and diversity with a number of environmental variables that characterise the investigated areas was assessed. According to the sampling strategy of the BIOFUN project, a total of six selected slopes were chosen along a gradient of increased oligotrophy in the three main Mediterranean basins; i.e., the western (WM; Algero-Provençal basin), central (CM; Ionian Sea) and eastern (EM; northern Levantine basin) Mediterranean basins (see Fig. 1). All of the selected open-slope systems were from topographically regular settings, with well-oxygenated bottom waters. Three of the slopes were in the WM basin (WM-1, Balearic slope 1; WM-2, Balearic slope 2; WM-3, Sardinia slope), two in the CM basin (CM-1, Maltese slope; CM-2, Ionian slope) and one in the EM basin (EM, Cretan slope) (Fig. 1). For each slope, three stations at three different depths were sampled. The three different station depths always fell into three depth ranges: upper bathyal (1200 m), mid-bathyal (from 1800 m to 1900 m), and lower bathyal (from 2400 m to 2700 m). CM-1 was not sampled at the lower bathyal, and was substituted by a station at a depth of 2120 m. At each station and with the employment of cylindrical box-corers (see details below), independent replicate samples were taken for the analyses of the macrobenthos (n = 3 replicates), microbial (n = 3 replicates) and environmental (n = 3 replicates) variables. We selected the heterogeneity of the substrate (grain size), the organic matter content of the sediments, and the prokaryotic abundance and biomass as recognized drivers that influence the benthic fauna distribution and diversity [10], [17]. The details of the sampling locations and the environmental features of all of the stations are given in Table 1 and Table S1.

**Table 1:** Main characteristics of the sampling slopes studied along the six Mediterranean continental slope areas.

Slope	Water depth (m)	Bottom temperature (°C)	Salinity	POC flux (mgC m^−2^ d^−1^)	Sand (%)	Silt (%)
**WM-1**	1224	13.1	38.5	18.2	9.0	91.0
**WM-1**	1803	13.2	38.5	11.3	13.8	86.2
**WM-1**	2362	13.3	38.5	9.0	12.3	87.7
**WM-2**	1179	13.1	38.5	12.7	6.3	93.7
**WM-2**	1862	13.2	38.5	8.8	8.9	91.1
**WM-2**	2758	13.3	38.5	7.4	6.3	93.7
**WM-3**	1258	13.3	38.5	12.2	8.0	92.0
**WM-3**	1890	13.2	38.5	8.2	14.1	85.9
**WM-3**	2448	13.3	38.5	6.1	18.7	81.3
**CM-1**	1236	13.7	38.7	22.1	3.3	90.5
**CM-1**	1798	13.7	38.7	14.7	1.3	98.7
**CM-1**	2120	13.8	38.7	10.9	2.6	97.4
**CM-2**	1219	13.7	38.6	13.4	14.3	85.7
**CM-2**	1924	13.7	38.6	6.6	39.9	60.2
**CM-2**	2693	13.8	38.6	4.6	33.7	66.3
**EM**	1237	14.7	38.8	11.0	3.6	96.4
**EM**	1907	14.7	38.8	7.0	10.1	89.9
**EM**	2766	14.7	38.7	5.1	3.0	97.0

WM, CM, EM, western, central, eastern Mediterranean.

POC, particle organic carbon.

### Environmental variable sampling

The near-bottom temperature and salinity were recorded, using a conductivity–temperature–depth (CTD) SBE 911 plus probe mounted on a CTD rosette system (Table 1). To analyse grain size and biochemical composition of the organic matter content, subsamples of sediments from each box-corer were collected using plexiglass cores of 3.6-cm internal diameter. As analysis of the top 1-cm layer has been shown to represent a feasible proxy for the whole trophic status of a sediment [61], [62], only the top 1-cm of subsamples was collected and frozen at −20°C, for the analysis of chlorophyll-a, phaeopigment and organic matter content.

### Grain size

The subsamples for the grain size analysis (the top 20 cm) were preserved at +4°C. Aliquots of fresh sediment were sieved over a 63-µm mesh. The two fractions (>63 µm, sand; <63 µm, silt and clay) were dried in an oven at 60°C and weighed. Data were expressed as percentages of the total sediment dry weight.

### Phytopigment contents and seafloor particulate organic carbon flux

Chlorophyll-a and phaepigments were determined according to standard tecniques [63]. The sum of the chlorophyll-a and phaeopigment concentrations were defined here as chloroplastic pigment equivalents (CPE). The concentrations of these total phytopigments were converted into carbon (C) equivalents using the conversion factor of 40 [64], and expressed as mgC g^−1^.

Johnson et al. [65] showed that the estimated particulate organic C (POC) flux from the surface represents a good predictor of the benthic standing stock, even at large spatial scales. We extracted the surface primary production data (as mgC m^−2^ d^−1^) from the ocean productivity database (http://www.science.oregonstate.edu/ocean.productivity/index.php). These data were used to estimate the flux of C to the seafloor, using Equation (1), as reported in Lutz et al. [66], and introduced by Suess [67]: 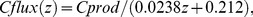
(1)where the C flux to depth C_flux_(z) is described as a function of the primary production of organic carbon in the surface waters C_prod_, scaled to the depth below the sea surface, z.

### Quantity and biochemical composition of the organic matter

The contents of carbohydrate, protein and lipid were determined according to standard techniques [63]. These concentrations were then converted into C equivalents using the conversion factors of 0.40, 0.49 and 0.75 µgC µg^−1^, respectively [63], and normalised to the sediment dry weight after desiccation (60°C, 24 h). Biopolymeric organic C (BPC) was calculated as the sum of the C equivalents of carbohydrate, protein and lipid [68]. The contributions of phytopigment C (CCPE) and protein C (CPRT) to the BPC concentrations (CCPE/BPC and CPRT/BPC ratios, respectively) and the protein/carbohydrate (PRT/CHO) ratio were then calculated and used as descriptors of the aging, origin and nutritional quality of the sediment organic matter [62]. PRT/CHO ratios >1.0 indicate relatively high quality and high food availability for the organisms [62].

### Prokaryotic abundance and biomass

For the analyses of the prokaryotic abundance and biomass, subsamples of sediments from each box-corer were collected using plexiglass cores of 3.6-cm internal diameter. *Circa* 1 ml of the wet surface sediment layer (0–1 cm) was fixed using buffered formaldehyde (2% final concentration, in sterile, filtered seawater [v/v]), and stored at 4°C until processed [69].

The total prokaryotic number (TPN) was determined using a staining technique with acridine orange [70], and analysed using epifluorescence microscopy (magnification, 1000×). The total prokaryotic biomass (TPB) was estimated using an ocular micrometer, assigning the prokaryotic cells into different size classes based on their maximum length and width [71]. These were converted to biovolumes on the assumption of an average C content of 310 fgC µm^−3^ [71]. The TPN and TPB were normalised to the sediment dry weight after desiccation (24 h, 60°C).

### Macrofaunal sampling

At each station, three independent replicates of undisturbed sediment samples were collected using cylindrical box-corers (Ø 50 cm, WM-2 and CM-2; Ø 32 cm, all other stations). From each box-corer sample, the top 20 cm of the sediment, along with their overlying water, were gently sieved over a 300-µm mesh sieve to retain all of the macrobenthic organisms [72]. The residual left behind on the sieve was immediately fixed in 10% buffered formalin solution, and stained with Rose Bengal.

### Macrofauna abundance, biomass and biodiversity estimation

All multicellular organisms (including Nematoda, Copepoda and Ostracoda; macrofauna *sensu lato*, [73], [74]) and Foraminifera that were retained on a 300-µm mesh sieve were sorted under a stereomicroscope, and identified to the lowest possible taxonomic level according to the main literature [59], [75]–[78]. The taxon names of the organisms were cross-checked with the World Register of Marine Species (WoRMS, www.marinespecies.org). For each species the total number was calculated and the wet-weight biomass measured; the number of individuals and weight were expressed as abundance and biomass per square meter. The wet biomass (g wet weight m^−2^) was converted to ash-free dry weight and organic carbon content using standard conversion factors [79]. In accordance with the literature [59], [75], [76]–[78], four major macrofaunal trophic (functional) groups were identified: surface deposit feeders (SDFs), subsurface deposit feeders (SSDFs), carnivores/scavangers, and filter feeders/suspension feeders.

Biodiversity was measured as α-diversity (or ‘sample’ diversity) by calculating several indices: species richness (SR), or total number of species collected in each boxcorer sample; Shannon-Weaner index (H′: log_2_) [80]; and Pielou's [81] index of equitability (J′). Moreover, the species-abundance data were converted into rarefaction diversity indices ([82], as modified by Hurlbert [83]), and the expected number of species ES(*n*) for theoretical samples of *n* = 30 and *n* = 50 individuals were calculated for each station. This method of rarefaction provides a good tool for comparisons of species richness among samples that have different total abundances [84]. The number of higher taxonomic groups identified (e.g., Polychaeta, Isopoda, Tanaidacea, Bivalvia, and others) in each of the samples was also considered. To characterise the macrobenthic community structure, the percentage contribution of each of the higher taxonomic groups to the total abundance and biomass was calculated.

The degree of change in the species composition between habitats or along an environmental gradient is usually defined as the turnover (β)-diversity. The macrofaunal turnover diversity between the different depths and longitudes was measured by the dissimilarity coefficients, based on a Bray-Curtis similarities matrix. The statistical differences in the macrofauna composition among all sampling sites was tested by the analysis of similarities (ANOSIM) [85].

### Statistical analyses

To test for differences in the patterns of environmental (i.e., temperature, salinity, grain size, quantity and quality of organic matter) and biological (i.e., macrofauna, microbial components) variables between different longitudes and stations at different depths, distance-based permutational multivariate analysis of variance was used (PERMANOVA; [86], [87]). The design included two factors: slope location (six levels, fixed, from the west to the east basin) and depth (three levels, fixed). The analysis was based on Euclidean distances of previously normalised data, using 999 random permutations of the appropriate units [88]. The tests were carried out using the permutation of residuals under a reduced model. As there was a restricted number of unique permutations in the pair-wise tests, the p values were obtained from Monte Carlo tests [89]. When significant differences were observed between stations at different longitudes and/or depths, pair-wise comparisons were also performed.

The β-diversity in the macrobenthic organism composition and trophic structure was estimated: (i) between basins; (ii) between stations at the same depth; (iii) between slopes within the same basin; and (iv) between stations at different depths. The turnover diversity was estimated through Bray-Curtis dissimilarity coefficients. The SIMPER analysis was used to identify the organisms that contributed the most to the dissimiarity between longitudes and depths.

To test for the presence of statistical differences in the macrobenthic organism compositions and functional structures, analysis of similarities (ANOSIM) was performed, as above: (i) between basins; (ii) between stations along the same depth; (iii) between slopes within the same basin; and (iv) between stations at different depths. All of the macrofaunal abundance data were presence/absence transformed prior to the analysis. When significant differences were observed, a non-metric multidimensional scaling ordination was carried out to visualise similarities between basins, slopes and depths along the same slope area. PERMANOVA, ANOSIM, SIMPER and nMDS analyses were performed using the PRIMER version 6 software package [90].

To determine whether the investigated environmental variables influence changes in the macrofaunal standing stock, trophic composition and diversity between basins and between slopes in the same basin, non-parametric multivariate multiple regression analysis was used, with the DISTLM forward routine [87]. The regression analysis was based on Euclidean distances when abundance, biomass and percentage of different trophic groups were considered, and on Bray-Curtis distances when diversity indices were tested. The forward selection of the predictor variables was carried out with tests by permutation. P- values were obtained using 4999 permutations of raw data for the marginal tests (tests of individual variables), while for all of the conditional tests, the routine used 4999 permutations of residuals under a reduced model. Bottom temperature, salinity and grain size were used as environmental parameters. BPC content, phytopigments content, microbial standing stock (i.e., abundance and biomass) and estimates of POC fluxes to the bottom were selected as indicators of food quantity, and the CCPE/BPC, CPRT/BPC, and PRT/CHO ratios as proxies for the quality of the sedimentary organic matter.

## Results

### Environmental features and trophic state of the sampling sites

The water mass features (temperature, salinity) and sediment grain sizes (sand, silt) are reported in Table 1. The bottom water temperature and salinity increased significantly moving eastwards (pair-wise tests; p<0.01), with values that ranged from 13.1°C and 38.5 for the WM basin to 14.7°C and 38.8 in the EM basin. The dissolved oxygen content ranged between 3.7 ml l^−1^ and 4.8 ml l^−1^, with the lowest concentration registered in the EM basin at 1200 m in depth, and the highest in the WM basin (WM-1) at 2400 m in depth. Most of the sediment was silt (Table 1, range, 60%–97.4%) at all depths and for all sites. A significantly higher percentage of silt fraction was seen for the CM-1 slope in the central basin, compared to those of the WM basin (pair-wise tests, p<0.01). The variability in the water mass and grain size occurred mainly at different longitudes, while there were no significant changes with depth (Table S2, PERMANOVA results). Significant changes in the quantity of the organic matter (Tables 1, 2, CPE, POC flux, BPC) were detectable at both different longitudes and different depths (Table S2, PERMANOVA tests). However, the organic matter content did not show any clear increasing or decreasing trends with depth or moving eastwards. The only exception was the POC flux, which clearly declined with increasing depth (pair-wise tests, p<0.01). Relatively high organic matter quantities characterised both the WM (WM-2) and CM (CM-1) basins, compared to the other slope systems (pair-wise tests, p<0.05). Significant changes in the organic matter quality (Table 2, CCPE, CPRT, PRT/CHO ratio) were detected at different longitudes, but not at different depths (Table S2, PERMANOVA tests). In particular, a high organic matter quality was seen for the sediments along the CM-1 slope in the CM basin (pair-wise tests, p<0.01). The prokaryotic standing stock (Table 2, abundance, biomass) varied significantly with longitude and depth (Table S2, PERMANOVA tests), although most of the variability was explained by the effect of longitude (see pseudo-F values, Table S2). The highest values for both the prokaryotic abundance and biomass were seen for the CM basin (i.e., CM-1 slope; pair-wise tests, p<0.01).

**Table 2:** Organic matter quantity, quality and prokaryotic standing stock.

Slope	Depth (m)	CPE (µg g^−1^)	CCPE/BPC (%)	BPC (mgC g^−1^)	CPRT/BPC (%)	PRT/CHO	TPN (ncell g^−1^)	TPB (µgC g^−1^)
WM-1	1224	0.42±0.18	2.26±0.96	0.73±0.03	25.15±7.38	0.40±0.19	4.06E+07±2.56E+06	0.81±0.05
	1803	0.62±0.01	3.84±0.48	0.66±0.06	23.24±0.71	0.31±0.04	2.53E+07±4.08E+06	0.65±0.13
	2362	0.60±0.09	3.70±0.67	0.65±0.02	17.04±3.32	0.19±0.04	2.85E+07±4.35E+06	0.57±0.09
WM-2	1179	6.19±0.18	11.70±0.77	2.13±0.11	35.30±1.31	0.67±0.07	1.30E+08±8.85E+06	3.46±0.57
	1862	1.98±0.22	6.51±0.51	1.21±0.04	36.68±3.55	0.78±0.15	6.94E+07±1.69E+06	2.82±0.60
	2758	2.71±0.50	8.82±2.07	1.25±0.08	37.54±4.94	0.96±0.21	1.08E+08±7.80E+06	3.94±0.81
WM-3	1258	0.71±0.04	3.72±0.11	0.67±0.10	42.67±8.08	1.11±0.40	8.71E+07±9.41E+06	1.74±0.19
	1890	1.12±0.08	6.86±1.18	0.68±0.07	34.13±11.40	0.51±0.18	8.67E+07±1.60E+07	1.73±0.32
	2448	0.96±0.17	5.50±1.31	0.80±0.29	40.79±5.70	1.02±0.33	5.94E+07±8.23E+06	1.19±0.16
CM-1	1236	4.16±0.34	10.50±1.70	1.64±0.19	50.10±1.41	3.68±0.08	1.64E+08±1.43E+07	5.33±0.26
	1798	8.35±0.27	23.75±1.01	1.41±0.05	54.86±2.05	2.92±0.21	1.82E+08±1.20E+07	7.61±0.13
	2120	1.63±0.15	5.95±0.72	1.11±0.07	56.32±1.86	2.20±0.32	3.01E+08±4.00E+07	10.58±0.85
CM-2	1219	1.83±0.19	8.59±0.97	0.85±0.01	28.11±2.68	0.46±0.05	8.45E+07±5.40E+06	1.69±0.11
	1924	2.63±0.69	14.89±4.26	0.72±0.03	39.89±3.37	0.80±0.15	1.60E+08±1.12E+07	3.20±0.22
	2693	1.73±0.80	8.64±4.23	0.82±0.03	21.03±2.58	0.29±0.04	1.82E+08±3.31E+07	3.65±0.66
EM	1237	1.96±0.17	10.34±0.85	0.76±0.03	22.74±3.57	0.26±0.05	5.49E+07±9.43E+06	1.15±0.21
	1907	0.42±0.00	1.93±0.05	0.87±0.03	24.84±1.54	0.40±0.03	8.05E+07±4.07E+06	2.15±0.13
	2766	0.55±0.08	2.20±0.32	1.01±0.04	22.32±0.42	0.31±0.01	1.42E+07±7.76E+05	0.39±0.07

Data are means ± standard deviation.

CPE, total phytopigments; CCPE/BPC, contribution of phytopigment C to biopolymeric C; BPC, biopolymeric C; CPRT/BPC, contribution of protein C to biopolymeric C; PRT/CHO, protein to carbohydrate ratio; TPN, total prokaryotic abundance; TPB, total prokaryotic biomass.

### Macrofauna abundance, biomass and community structure

**Figure 2:**
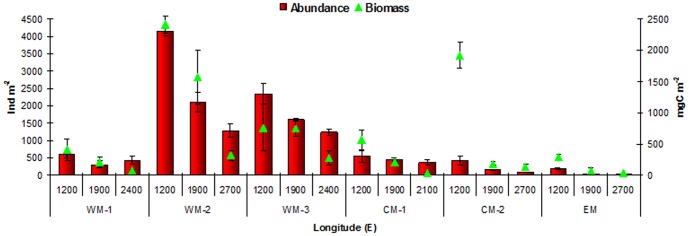
Macrofaunal standing stock. Mean total macrofauna abundance (bars; ind/m^2^) and biomass (triangles; mgC/m^2^) for each station in the WM, CM and EM basins. Data are means ± standard deviation (n = 3).

**Figure 3:**
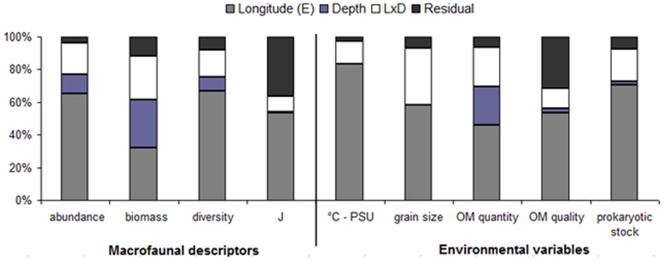
PERMANOVA results. Contributions of the components of variance (longitude, depth, longitude × depth [L×D]) according to the macrofaunal descriptors and the main environmental features. Diversity, macrofaunal diversity (SR, H′:log2, Hulbert index); Pielou evenness (J′); bottom temperature (°C); bottom salinity; organic matter (OM); and prokaryotic stock.

The total macrofaunal abundance and biomass are shown in Figure 2 and reported in Table 3. Significantly higher values for the macrobenthic standing stock were seen for two of the slope areas in the WM basin: WM-2 and WM-3 (pair-wise tests, p<0.01, vs. all of the other slopes) Differences in the standing stock with depth were generally seen for all of the slope areas between the shallower stations (1200 m) and the deeper stations (pair-wise tests, p<0.05), except for the WM-1 slope area, where no significant differences between the depths were detected. The PERMANOVA tests carried out on the macrofaunal biomass and abundance showed significant differences according to both longitude and depth (Table S1). Longitude explained most of the variability in the macrofaunal abundance (65%), while both longitude and depth explained the variability in the macrofaunal biomass (32% and 30%, respectively) (Fig. 3).

**Table 3:** Macrofaunal variables.

Slope	Depth (m)	Abundance (Ind m^−2^)	Biomass (mg m^−2^ WW)	Structural diversity					
				N° taxa	SR	ES(30)	ES(50)	Shannon H′	Pielou J′
WM-1	1224	600±102	405.9±170.6	8±1	59±5	11±2	19±2	4.17±0.27	0.71±0.01
	1803	314±104	209.8±77.3	6±1	60±3	13±1	24±1	4.90±0.20	0.83±0.01
	2362	434±103	72.5±8.5	5±0	46±1	8±4	16±2	3.22±0.66	0.58±0.16
WM-2	1179	4153±128	2412.8±141.4	16±1	148±3	14±1	25±1	5.25±0.14	0.73±0.02
	1862	2104±287	1567.1±432.8	13±1	96±5	13±2	25±1	5.04±0.17	0.77±0.02
	2758	1281±204	321.9±81.5	14±1	74±3	12±1	21±0	4.48±0.12	0.72±0.02
WM-3	1258	2349±300	765.2±371.1	12±0	97±4	11±1	21±1	4.54±0.14	0.69±0.01
	1890	1615±45	752.1±127.2	10±0	77±2	10±1	18±1	4.13±0.13	0.66±0.02
	2448	1248±88	274.7±106.5	8±1	63±1	8±1	15±1	3.27±0.16	0.55±0.03
CM-1	1236	556±180	565.6±163.9	10±1	51±7	14±4	26±4	5.07±0.44	0.89±0.01
	1798	452±41	207.0±13.2	9±1	46±2	14±1	24±1	4.85±0.14	0.88±0.00
	2120	365±83	40.0±11.9	8±1	37±3	12±2	21±2	4.37±0.27	0.84±0.03
CM-2	1219	438±133	1926.0±215.9	8±1	51±3	14±1	24±2	4.88±0.19	0.86±0.01
	1924	145±13	186.7±27.3	9±0	24±1	10±1	17±1	3.80±0.16	0.83±0.01
	2693	84±4	137.5±47.9	5±0	17±1	10±2	15±1	3.62±0.19	0.89±0.01
EM	1237	199±25	284.7±59.2	4±1	20±0	10±1	16±0	3.71±0.14	0.86±0.03
	1907	37±19	72.5±53.0	2±0	4±0	4±1	-	1.66±0.33	0.83±0.00
	2766	33±8	37.9±16.1	2±0	7±1	7±0	-	2.75±0.32	0.98±0.03

Data are means ± standard deviation.

WW, wet weight; SR, species richness; ES(30), ES(50), Hulbert indices.

**Figure 4:**
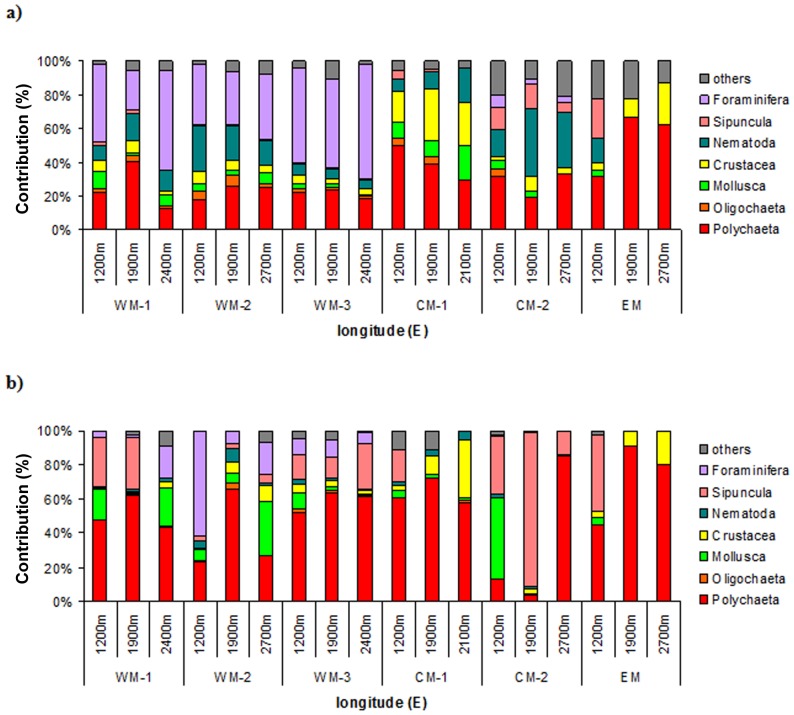
Macrobenthic community structure. Macrofaunal community structure in terms of (a) abundance contribution (%), and (b) biomass contribution (%) of the major taxonomic groups represented in the graphs as Polychaeta, Oligochaeta, Mollusca, Crustacea, Nematoda, Sipuncula, Foraminifera and others, at all of the investigated stations. The group of ‘others’ includes: Nemertea, Caudofoveata, Porifera, Bryozoa, Hydrozoa, Scyphozoa, Priapulida and Echiura.

A total of 22 higher taxa were identified (i.e., Foraminifera, Porifera, Hydrozoa, Scyphozoa, Nematoda, Nemertea, Oligochaeta, Polychaeta, Priapulida, Sipuncula, Echiura, Ostracoda, Copepoda, Cumacea, Tanaidacea, Isopoda, Amphipoda, Aplacophora, Scaphopoda, Gastropoda, Bivalvia, Bryozoa), with the highest mean number present of 16 seen for the WM-2 slope (Table 3). The different contributions in terms of the abundance and biomass of the most represented groups are reported in Figure 4 (grouped as Polychaeta, Oligochaeta, Crustacea, Mollusca, Nematoda, Sipuncula, Foraminifera, and others), for all of the slopes investigated. There were clear changes in the community compositions between the slopes at different longitudes, in terms of both abundance (Fig. 4a) and biomass (Fig. 4b). While the WM basin slopes were dominated by a high number of Foraminifera (range, 23%–67%), these were almost completely absent in the other Mediterranean basins. Polychaeta were relatively important in all of the stations, and in EM and CM-1, they were the dominant group (range, 31%–67%). Mollusca (i.e., mostly bivalves) were always at relatively low levels, and these peaked for the Maltese slope (CM-1), with a range of 9% to 21%. Sipuncula (range, 6%–14%), and particularly the macrobenthic Nematoda (range, 16%–40%), showed relatively high abundance along the CM-2 slope; the first group also had a relatively high abundance at the shallowest station, of EM (23%). Crustacea had the highest relative abundance at the CM-1 slope (range, 18%–30%; mostly Isopoda and Tanaidacea) and at the deepest station, of EM (Amphipoda, Ostracoda; 25%). Following these above-mentioned groups, Hydrozoa was the only other group of importance, but only for the EM slope, with a range of 12%–22%.

Polychaeta contributed the highest biomass for almost all of the stations (Fig. 4b; range, 13%–91%). The four exceptions (Fig. 4b) were: the WM-2 1200-m-deep station, with 61% of the biomass formed by branched Foraminifera; the WM-2 2700-m-deep station, with the bivalve *Nucula* sp.1 forming 31% of the biomass; the CM-2 1200-m-deep station, with the bivalve *Cuspidaria* sp.1 forming 47% of the biomass; and the CM-2 1900-m-deep station, with 90% of the biomass formed by Sipuncula (*Golfingia spp.* and *Phascolosoma spp*.) (Fig. 4b).

There was a significant difference in the community structure among the Mediterranean basins (ANOSIM, p = 0.001), but not between the depths. The Bray-Curtis coefficient of dissimilarity detected major changes in the community composition between the WM and CM basins (32%) and between the WM and EM basins (42%). In terms of the biomass contributions, again, significant differences were detected between the WM and CM basins (ANOSIM, p<0.05; dissimilarity coefficient 29%) and between the WM and EM basins (ANOSIM, p<0.01; dissimilarity coefficient 39%), without any differences across the depths.

### Macrofaunal α-diversity and trophic composition

The macrofaunal diversity indices are reported in Table 3. A total of 274 macrobenthic organisms were identified (Table S3). Significantly lower macrofaunal α-diversity was reported from the EM basin compared to the WM and CM basins (pair-wise tests, p<0.01). Along all of the slopes, with the sole exception of WM-1, the diversity decreased with depth, and significant differences were detected mostly between the 1200-m-deep stations and the deeper stations (pair-wise tests, p<0.05; Table 3). The macrofaunal α-diversity varied mostly with longitude (67%), and to a lesser degree with depth (8%) (see Fig. 3; Table S2). The variability in the equitability index J′ (Fig. 3; Table S2) was along the west-east axis, with significantly higher values for the CM and EM basin slopes compared to the WM basin slopes (pair-wise tests, p<0.01), which is converse to the result for the α-diversity.

**Figure 5:**
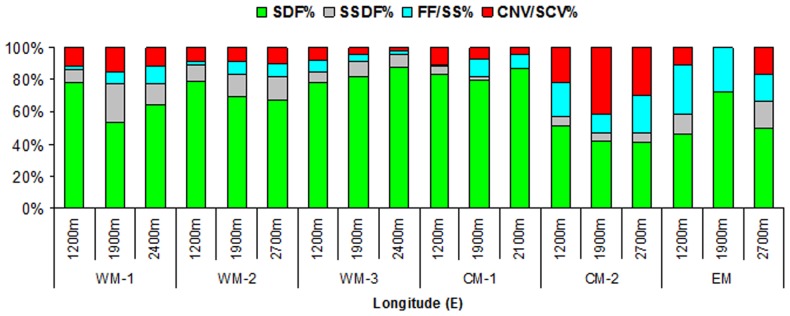
Macrofaunal functional composition. Trophic structure composition as the percentage contribution of each trophic group, from the WM basin to the EM basin and along all of the investigated slope areas. SDF, surface deposit feeder; SSDF, subsurface deposit feeder; FF/SF, filter feeder/suspension feeder; CNV/SCV, carnivore/scavanger; WM, western Mediterranean; CM, central Mediterranean; EM, eastern Mediterranean.

The trophic composition of the macrofauna is shown in Figure 5, i.e., for the four major functional groups which we considered. The surface deposit feeders (SDFs), which were mainly represented by Crustacea and Polychaeta, were dominant at all depths and for all of the areas (always >40%). The contribution of the subsurface deposit feeders (SSDFs), such as the Polychaeta of the families Capitellidae, Fauveliopsidae and Cossuridae, to total abundance decreased moving eastwards (range, 24%–5%), except for the 2700-m-deep station in the EM basin, which did not fit this trend. Carnivores (range, 5%–40%) had a peak in CM-2, which was mainly caused by Polychaeta of the families Eunicidae, Syllidae and Glyceridae, and Nematoda of the genera *Pareurystomina*, *Oncholaimellus*, *Trissonchulus* and *Pheronus.* Filter feeders were more abundant for CM-2 and EM (range, 8%–30%), which was due to small Hydrozoa (Fig. 5). Significant differences in the trophic structure compositions were detected along the longitudinal axis (Table S4), but not between different depths. Indeed, high similarities between the stations at increasing water depths were detected by the SIMPER analysis, which characterised each of the slope areas (range, 77%–96%, in the EM basin and along WM-2). There were major changes in the trophic compositions between the WM basin and the CM and EM basins. Pair-wise comparisons of the WM and CM basins and the WM and EM basins were highly significant, whereas those for the CM and EM basins were not (p = 0.16).

### β-diversity in the macrofaunal composition: longitudinal and bathymetric trends

Significant differences in β-diversity were found between: (i) the basins (Table S5A); (ii) the slopes of the different basins (Table S5B); and (iii) the slopes within each basin (Table S5C).

**Figure 6:**
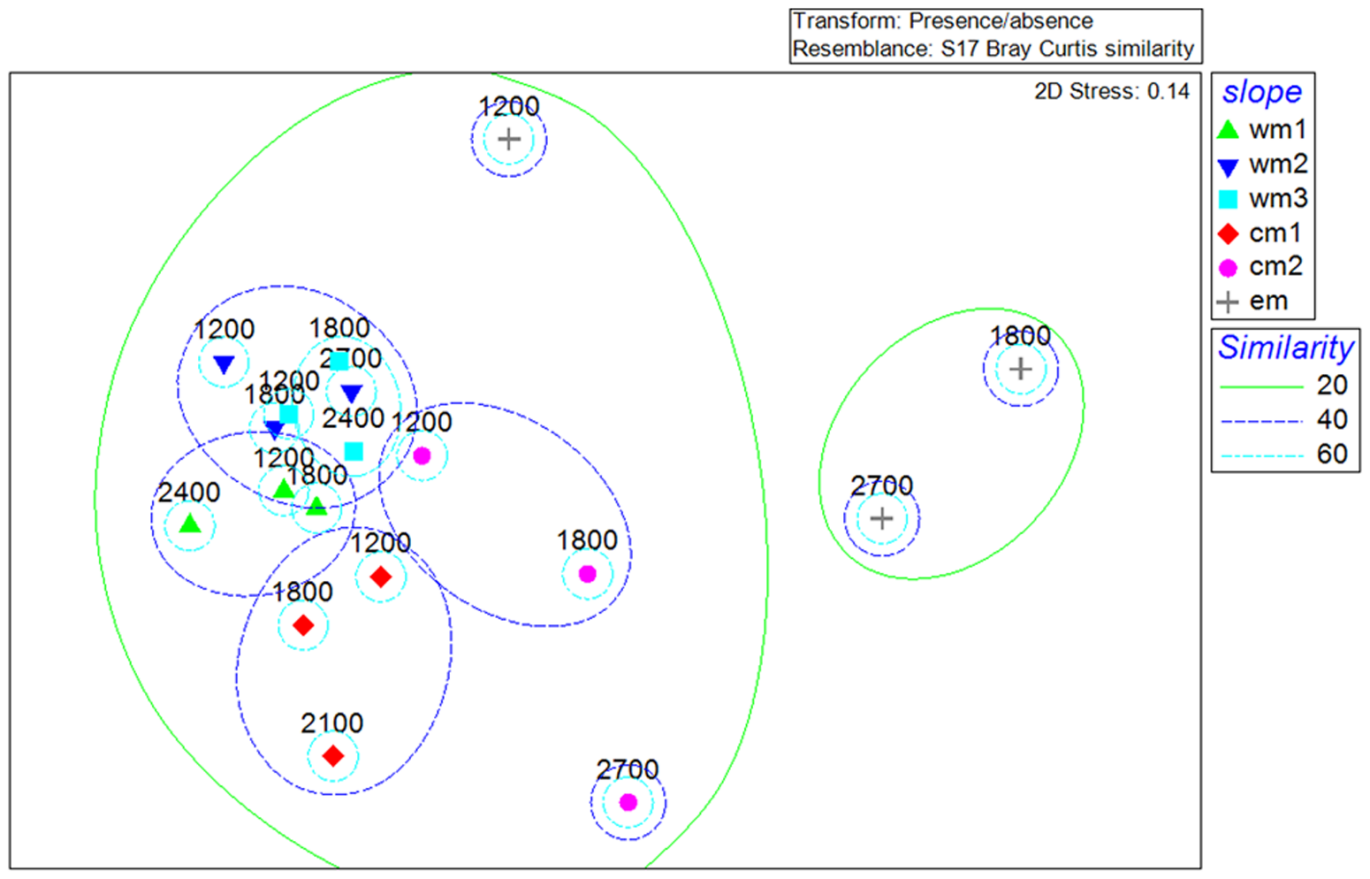
Non-metric multidimensional scaling. Non-metric multidimensional scaling ordination plot based on macrobenthic organisms composition, showing the similarity among the slopes at different longitudes and stations at different depths. wm1, wm2, wm3, WM basin; cm1, cm2, CM basin; em, EM basin. Numbers above symbols indicated station depths.

The macrobenthic community composition changed significantly also considering stations at different water depths within the same slope system. The main differences were observed between the communities inhabiting the upper bathyal stations and the mid- and deep bathyal stations (Table S6). A high β-diversity emerged at all levels, expressed by the coefficient of dissimilarity values: between basins (from 65% to 82%); between slopes (from 61% to 85%); between slopes within each basin (from 43% to 71%) (Table S5); and between stations at different depths (from 39% to 98%) (Table S6). The largest dissimilarities were seen when the EM basin communities were compared to those of the WM and CM basins. The overall dissimilarity between depths and basins was driven by small contributions of many species (Tables S7A, S7B). In the case of dissimilarities between basins, these species belonged mostly to Polychaete families, such as Maldanidae sp1 (1.85%; WM-CM basins), Cirratulidae sp1, and Spionidae sp1 (both 1.95%; WM and EM basins) and Paraonidae sp1 (3.49%; CM and EM basins). The dissimilarities between stations at different depths along the slopes were driven by organisms that belonged to different higher taxa, such as Bivalvia, Sipuncula, Foraminifera, Polychaeta and Tanaidacea, depending on the slope system considerered. The highest contribution came from *Golfingia* sp1, which drove the dissimilarity along the EM slope between both the 1200 m to 1900 m stations (11.09%) and the 1200 m to 2400 m stations (10.69%), which was absent in the two deeper stations. The high rates of turnover diversity were also evident in the nMDS (Fig. 6), which grouped slopes according to longitude and, to a lesser extent, according to depth, at a similarity of only 40% and 20%, respectively. In a multi-dimensional scaling representation, it emerged that slopes at different longitudes and stations at different depths differed in macrofaunal composition. Only a few organisms, 19 of a total of 274 organisms identified (i.e., 7%), were reported for all of the basins (Table S3). Most of these were Polychaeta (such as *Glycera* sp1; *Capitellidae* sp1; *Syllidae* sp1 or *Heterospionidae* sp1), Crustacea (Copepoda; the amphipods *Eusiridae* sp1 and Ostracoda), Nematoda (*Linhystera* sp1 and *Bathyeurystomina* sp1) and Sipuncula (such as *Golfingia* sp1). The density of these organisms changed from one basin to another, with them being widely represented in one or two basins and less represented in the others. Usually, they reached very low abundances in the EM basin. However, one explanation for this may be that the EM basin was under-sampled compared to the other basins.

### Macrofauna relationships with environmental variables

**Figure 7:**
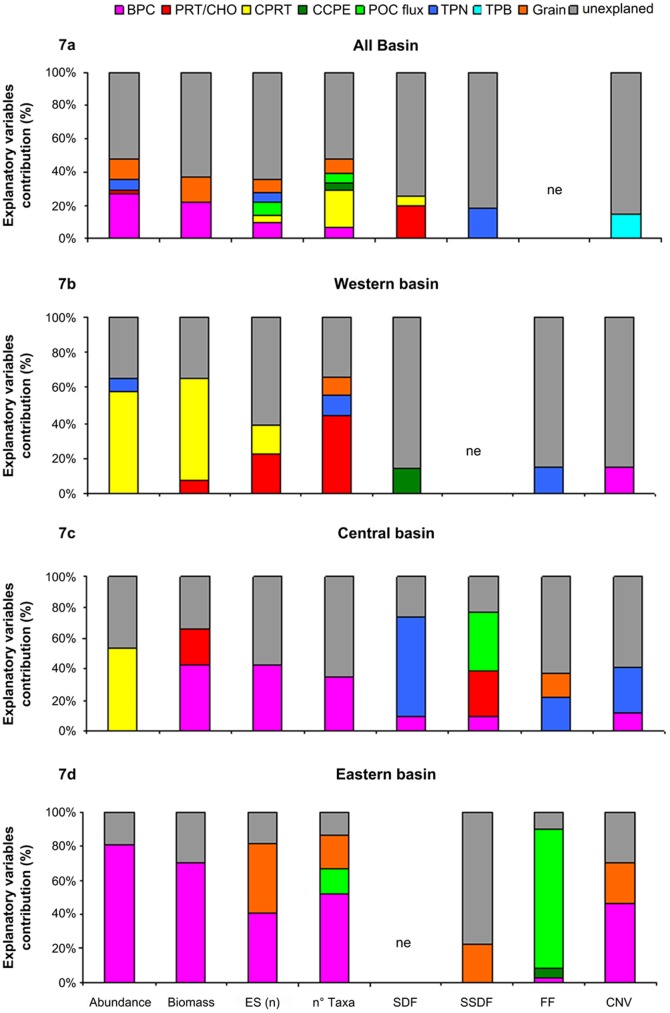
DISTLM forward results. Results of multivariate multiple regression analysis of the macrofaunal abundance, biomass, expected species number (ES(30, 50)), number of taxa, trophic community structure (%SDFs, %SSDFs, %FF/SF, %CNV/SCV), for (a) all of the investigated areas, and for the (b) WM, (c) CM, and (d) EM basins. The contributions are shown for the significant environmental variables (i.e., the explanatory variables) according to the variability of each of the macrofauna descriptors. BPC, biopolymeric organic C; PRT/CHO, protein/carbohydrate ratio; CPRT, protein C; CCPE, phytopigment C; POC, particulate organic C; TPN, total prokaryotic number; TPB, total prokaryotic biomass; Grain, grain size; ne, not explained.

To determine whether and how the environmental features and the trophic state of the system might influence the variability in the macrobenthic communities, multivariate multiple regression analysis (DISTLM forward) was carried out. These data are reported in Figure 7a and Table S8, for all of the slopes investigated, and in Figure 7b–d and Table S9 for each basin separately. Overall the most important factors that influenced the variability of the macrofauna abundance and biomass according to the above-mentioned analyses appeared to be the quantity of the food sources (BPC, 27%; TPN, 6%) and the heterogeneity of the substrate (grain size, 13%; Fig. 7a). The macrofaunal diversity appeared to correlate well not only with the quantity, but also with the quality (i.e., CPRT) of the organic matter in the sediment, and secondly with the grain size. The trophic compositions of the macrobenthic communities were only weakly influenced (range, 15%–26%) by the trophic sources, expressed as the microbial stock and the quality of food (Table S8). However, the high percentages of the variability in abundance, biomass and diversity did not correlate with our environmental variables (Fig. 7, grey).

As the trophic state of the system changed between the basins along the west-east axis, DISTLM forward analyses were also carried out considering each basin separately. Indeed, different drivers might be involved in the variance of the macrofauna distribution and diversity within each basin, compared to the data obtained for the whole of the Mediterranean Sea. Along the WM basin slopes, the macrofauna standing stock and diversity correlated with the quality of the organic matter (i.e., CPRT, PRT/CHO ratio), and to a lesser extent with the microbial abundance (range, 8%–11%) and the grain size (11%) (Fig. 7b; Table S9). Considering the available food, the quantity (i.e., TPN, BPC) and quality (i.e., CCPE) contributed significantly to the variance in the macrofaunal trophic groups, although overall they accounted for a limited amount of the variance (see Fig. 7b; Table S9).

In the CM basin, both the quantity (BPC) and quality (CCPRT) of the putative food sources might be drivers for the macrofauna standing stock and diversity variability (Fig. 7c; Table S9). The prokaryotic abundance in the sediment and the POC flux to the sea bottom were highly significantly correlated with the trophic group variability.

In the EM basin, the variance of the macrofaunal stock appeared to be influenced by the quantity of organic matter in the sediment (i.e., BPC). The diversity was correlated to the BPC content and to the heterogeneity of the substrate (Fig. 7d; Table S9). Once again, with the only exception being the SDFs, the variability of the different functional groups was related to the quantity of the available food sources (i.e., BPC, POC flux), and the grain size.

## Discussion

### Longitudinal and bathymetric trends in the macrofauna abundance, biomass, community and trophic structure

For the deep Mediterranean macrobenthos, there are no comparable datasets in terms of the spatial scale, because almost all of the available information is scattered and often restricted to specific areas (e.g., canyons or a single slope system). Since the investigations conducted in the EM basin from 1989 to 1998 across the continental shelf and at various bathyal depths [4], [32], [55], [91], and the limited set of data for the WM basin collected between 1988 and 1996 and in 2007 [23], [26], [58], there have been no other more recent studies on the deep Mediterranean macrobenthic infauna. Another point is that sediment has been sieved through mesh sizes ranging from 250 µm to 500 µm for the deep macrofauna. In effect, the discussion among European scientists in terms of what mesh size to use is still open, as studies in different countries use different mesh sizes [6]. This makes it difficult to directly compare quantitative data on deep-sea macrobenthic fauna from the Mediterranean Sea, and also worldwide.

Previous research within the Mediterranean Sea that included open slope systems has reported a general decline in the abundance and biomass of the benthic fauna (i.e. meiofauna, macrofauna, megafauna) with increasing depth and longitude [4], [36], [92]–[94]. One of the main causes of this decreasing trend in macrobenthic standing stock is the increasing oligotrophy of the water masses from the west to the east Mediterranean basin [92], [98]. The present study also recorded a decreasing trend in the macrofauna standing stock from the WM to the CM and EM basins, the only exception being the evident drop along the Balearic slope area (WM-1). Similarly, Tselepides et al. [95] reported lower values for the bathyal meiofaunal abundance from the south Balearic Islands area. They suggested that the paucity in the meiobenthic population can be ascribed to the oligotrophy of that area, which is influenced by the food-depleted Liguro-Provençal current. This observation is consistent with the general lower values for the food sources in the present study, which includes the microbial component in WM-1 relatively to WM-2 and WM-3. We can infer that the quantity and quality of the food (expressed as the BPC content) influence the macrobenthic population, as already shown for the Mediterranean Sea and for oceans worldwide [93], [99], [100], as well as for other benthic components (i.e., megafauna, meiofauna, prokaryotes) [28], [95], [101].

In the present study, an increase in the water depth was associated with a decrease in the macrobenthic stock, especially in the biomass. Similarly, other studies have reported sharper decreases with depth in the biomass rather than in the number of macrofauna organisms [12], [32], which appears to be mainly due to the rapid depletion of the food sources [96], [97].

The macrofaunal community composition changed with longitude rather than with depth, such that every slope system is a naturally heterogeneous system, including its fauna population [102]. The group of Polychaeta was not always the most abundant, in contrast to what has been reported in other studies [4], [32], [57]. In the WM basin, Foraminifera such as *Hoeoglundina elegans, Uvigerina mediterraean, Ammolagena clavata* and *Truncorotalia sp*. were present in higher levels (range, 23%–67%). Rosso and co-authors [103] found that some of these Foraminifera species were associated with deep-water corals from Santa Maria di Leuca (Italy); however, most other studies conducted in the Mediterranean Sea have excluded this group in macrobenthic studies [58], [103]. However, the Foraminifera can be an abundant and widespread component of deep-sea benthic populations, and they cover specific functional roles [104]–[107].

The other abundant groups found, such as Crustacea, Bivalvia, macrobenthic Nematoda and Sipuncula, have often been reported as important and diversified [34], [49], [50], [53] components of the Mediterranean bathyal fauna [4], [58], and of other seas [108], [109]. The identified Amphipoda families (e.g., Eusiridae, Phoxocephalidae, Lyssianassidae) and the species *Harpinia truncata* and *Paracentromedon crenulatum* usually inhabit the WM basin and the EM basin [51], [53], as well as for the Cumacea genera and the species recognized (e.g., *Cyclaspis longicaudata, Diastyloides bacescoi, Diastyloides serratus*) [49], [50], [52], [54]. A large contribution of Sipuncula, particularly in terms of biomass, was also documented by Cosson et al. [25] in their comparison of stations at increasing oligotrophy in the Atlantic Ocean. The Sipuncula can catch and bury food deeper in the sediment, in this way they can cope with conditions of low food sources [110]. Nematoda occurred widely from the WM basin to the EM basin slope areas. This is another group that has rarely been included in macrofauna studies [4], [26], [111], because it is considered an exclusively meiofaunal taxon [18]. They cover different functional roles and represent an important and distinct assemblage in the macrobenthos [111] and in comparison with nematodes from meiofauna. Indeed, with the sole exception of the highly represented genus *Halalaimus* also in our samples, the most abundant meiobenthic nematode genera reported by Pape et al. [29] (e.g. *Acantholaimus, Amphimonistrella, Monhystrella, Neochromadora*) were rarely or never found in our macrobenthic samples.

The trophic structure of a population can provide information on the trophic status of a system, and on the structural complexity of a community [32]. The dominance of SDF, followed by SSDF, for all of our slopes and depths confirms that the deposit feeding mode is one of the best feeding strategies in environments that generally have low food sources, such as the deep sea [105], [112]. Major changes in the trophic structure composition occurred moving eastwards, where the contributions of carnivores/scavengers and filter feeders gained importance, as reported previously [4], [32]. The carnivore/scavenger feeding mode is considered advantageous in a nutrient-limited environment, as their mobility is necessary to locate the more scarce food sources [4], [114]. The number of carnivores in our study substantially increased by including the many predatory/omnivore genera of large-sized Nematoda, as has been documented in other areas where food is relatively scarce [111], [115]. The relatively high percentage of filter feeders/suspension feeders in the EM basin, which was also reported by Tselepides et al. [32], was explained by Kröncke et al. [4] in terms of the large-scale hydrodynamic features of the open basin (e.g., lateral transport of organic material from coastal regions). The link between the filter feeders/suspension feeders group and the organic carbon fluxes was confirmed by the strong correlation observed between these filter feeders/suspension feeders and the POC flux in the EM slope area.

Overall, the data in the present study show that the trophic diversity of the Mediterranean macrobenthic populations might be influenced only partially by food availability and the heterogeneity of the substrate (range, 15% to 26%). However, it has been noted [22], [105] that correlations between food availability and feeding strategies of benthic organisms might be more the result of a combination of factors (e.g. hydrodynamic conditions, small-scale physical events), which can influence the availability of food sources for the benthic populations. Even though the communities in the WM basin appeared to be less affected by food availability, this appears to have an important role in the CM and EM basins for the determination of the functional structure of the macrofauna [55], [58]. In the CM basin, the highest microbial and organic matter qualities were reported, which may point to the influence of the available food on the macrofaunal trophic structure, and in particular, of the grazing activity of the macrofauna on the microbial organisms [113].

### Macrofaunal α-diversity versus β-diversity

For the Mediterranean Sea, the species diversity from meiofauna to megafauna has been reported to show an overall decline both with increasing water depth and with longitude, even though some exceptions have been observed [98]. Patterns in the faunal biodiversity are usually ascribed to a decrease in food availability with increasing water depth, and in the case of the Mediterranean Sea, also with an eastwards trend in food sources [20].

Our data confirm the longitudinal decreasing trend in macrofaunal species richness from west to east and with depth for the Mediterranean Sea, along all of the slopes investigated. The food availability and heterogeneity of the substrate appear to influence the diversity of the macrofauna (i.e., ES(30, 50), number of taxa), especially when the effects of these variables are tested within each basin of the Mediterranean Sea. The potential drivers that are usually mentioned to explain patterns in faunal biodiversity (i.e., food availability, sediment grain size) have important roles also in the present study, particularly at a within-basin spatial scale. Indeed, some drivers act differently, but simultaneously, on smaller or larger spatial scales, where they might often be hidden by the effects of depth, longitude and/or latitude [8], [84], [116], [117]. The equitability index (J) showed an opposite trend to that of the diversity indices. This means that moving eastwards, the dominance of some Foraminifera (e.g., *Uvigerina mediterranea*) and Polychaeta (e.g., Cirratulidae, Fauveliopsidae) disappeared.

It has been demonstrated that a simple analysis of local α-diversity is not enough to evaluate the biogeographic differences in deep-sea species compositions, and therefore does not provide a real picture of the biodiversity that characterises different systems, as well as the factors that control them [85], [118]. Here we have quantified for the first time the deep Mediterranean basin β-diversity of macrofauna across different depths and longitudes. While the α-diversity showed significant differences between the WM, CM and EM basins and some variability along the bathymetric gradient, the β-diversity revealed large changes in the macrobenthic organism compositions at the different levels: (i) between the basins and slopes; (ii) between the slopes within the same basin; and (iii) at different depths. No clear spatial overlap emerged between slope systems or depths. In contrast to what was reported by Vanreusel et al. [119], but similar to the findings of Serpetti et al. [109], the organisms that generated the high rates of turnover diversity were not necessarily organisms that were dominant along one slope or at one particular depth. The low overlap in the species compositions can be ascribed to the high habitat heterogeneity that is typically reported for the continental margins [19]. The reasons for this heterogeneity might include differences in food supply, substrate heterogeneity, hydrological features and/or geographic position [8].

The high macrofauna β-diversity is comparable to that reported for nematodes [31], [120] and for megafauna [36], which demonstrates a highly variable macrofauna composition at different longitudes and depths. For this reason, we can hypothesise that macrofaunal biodiversity is determined locally (i.e., on smaller spatial scales), and even more, regionally (i.e., on larger spatial scales). This is in agreement with findings reported for other benthic compartments, and it indicates that each region in the Mediterranean Sea can be distinguished according to the presence of a specific assemblage and species composition.

## Conclusions

From our large spatial scale investigation of the macrofauna that inhabit the deep Mediterranean Sea, it has emerged that:

- The macrobenthic abundance and biomass show a general longitudinal decreasing trend from the WM basin to the EM basin. Biomass, rather than abundance, is negatively affected by increasing water depth;
- The macrobenthic community and trophic structure change significantly with longitude; there were no significant changes here between depths;
- The macrofaunal standing stock, diversity and trophic structure are differently influenced by the quantity and quality of the food sources and the habitat features (e.g., grain size), which depend on the basin or slope system investigated. From our analysis, we can infer that the influence of the food source or substrate heterogeneity on the benthic fauna might be modulated or partially masked by the multiplicity of interactions between ‘local’ ecological characteristics and environmental factors, as opposed to those considered here, for each specific basin and/or slope environment;
- The high β-diversity through the Mediterranean basins and for different depths suggests notable large (i.e., between basins) and smaller (i.e., across depths along the same slope system) spatial scale diversity in the macrofauna composition that is not detectable by estimating the α-diversity alone.

The present study also highlighted the following gaps in the study of the deep Mediterranean Sea macrofauna:

- A lack of recent comparable datasets (e.g., for standing stock, α-diversity, β-diversity) on large spatial scales;
- A lack of a unified sampling technique for the macrobenthos;
- The *a-priori* exclusion of some organisms, such as macrobenthic Foraminifera and Nematoda, even though they often constitute an important and distinct component of the macrobenthos, in terms of their abundance, biomass, and structural and functional diversity.

## Supporting Information

Table S1 Sampling details.
(DOC)

Table S2 PERMANOVA results carried out to ascertain multivariate differences in environmental features, prokaryotes and macrofauna at different longitudes and depths.
(DOC)

Table S3 List of the identified macrobenthic organisms.
(DOC)

Table S4 Dissimilarity in trophic groups composition between basins and variables responsible for the estimated differences.
(DOC)

Table S5 Dissimilarities in macrobenthic organisms composition between all investigated A) basins; B) slopes; C) slopes within each basin.
(DOC)

Table S6 Dissimilarities in the macrofaunal organisms composition between depths at all investigated slopes, from west to east basin.
(DOC)

Table S7 A) Contribution of macrobenthic organisms responsible for the dissimilarity between depths. B) Contribution of macrobenthic organisms responsible for the dissimilarity between basin.
(DOC)

Table S8 Results of the multivariate multiple regression analysis carried out on the macrofaunal descriptors.
(DOC)

Table S9 Results of the multivariate multiple regression analysis carried out separately in the western, central and eastern basins on the macrofaunal descriptors.
(DOC)
