# Supplementary material for: Large Spatial Scale Variability in Bathyal Macrobenthos Abundance, Biomass, α- and β-Diversity along the Mediterranean Continental Margin
Source: PLoS One. 2014 Sep 16;9(9):e107261. doi: 10.1371/journal.pone.0107261 (PMC4165892; doi:10.1371/journal.pone.0107261)
Supplement: Table S1 — Sampling details. (DOC) [file pone.0107261.s001.doc]

**Table S1.** Sampling details.

| **Basin** | **Period** | **Station** | **Lat (°N)** | **Long (°E)** | **Depth (m)** | **R/V** | **Surf. sampled (m^2^)** |
| --- | --- | --- | --- | --- | --- | --- | --- |
| WM | May/2010 | WM-1 | 39.2709 | 3.3883 | 1224 | Urania | 0.241 |
|  |  |  | 39.2026 | 3.4336 | 1803 | Urania | 0.241 |
|  |  |  | 38.9398 | 3.6238 | 2362 | Urania | 0.241 |
| WM | Nov/2009 | WM-2 | 39.6007 | 4.1454 | 1179 | Pelagia | 0.589 |
|  |  |  | 39.2500 | 4.1778 | 1862 | Pelagia | 0.589 |
|  |  |  | 39.2289 | 5.4191 | 2758 | Pelagia | 0.589 |
| WM | Oct/2009 | WM-3 | 40.5450 | 7.6980 | 1258 | Urania | 0.241 |
|  |  |  | 40.5474 | 7.6730 | 1890 | Urania | 0.241 |
|  |  |  | 40.5555 | 7.6448 | 2448 | Urania | 0.241 |
| CM | May/2009 | CM-1 | 36.4314 | 15.5181 | 1236 | Urania | 0.241 |
|  |  |  | 36.4220 | 15.5462 | 1798 | Urania | 0.241 |
|  |  |  | 36.4167 | 15.5891 | 2120 | Urania | 0.241 |
| CM | Dec/2010 | CM-2 | 34.5291 | 16.3428 | 1219 | Meteor | 0.589 |
|  |  |  | 34.5187 | 16.8077 | 1924 | Meteor | 0.589 |
|  |  |  | 34.5986 | 17.3629 | 2693 | Meteor | 0.589 |
| EM | Jun/2008 | EM | 34.9539 | 24.5709 | 1237 | Urania | 0.241 |
|  |  |  | 34.8831 | 24.5471 | 1907 | Urania | 0.241 |
|  |  |  | 34.6388 | 24.3487 | 2766 | Urania | 0.241 |

Indicated are: the basin of origin (WM: western Mediterranean; CM: central Mediterranean; EM: eastern Mediterranean); the period of sampling (month/year); station code (indicating basin and slope); geographical position (Latitude N, Longitude E); water depth; research vessel (R/V); total area of sediment sampled for the macrofauna analyses (Surface sampled).
