# Supplementary material for: Large Spatial Scale Variability in Bathyal Macrobenthos Abundance, Biomass, α- and β-Diversity along the Mediterranean Continental Margin
Source: PLoS One. 2014 Sep 16;9(9):e107261. doi: 10.1371/journal.pone.0107261 (PMC4165892; doi:10.1371/journal.pone.0107261)
Supplement: Table S2 — PERMANOVA results carried out to ascertain multivariate differences in environmental features, prokaryotes and macrofauna at different longitudes and depths. (DOC) [file pone.0107261.s002.doc]

**Table S2.** PERMANOVA results carried out to ascertain multivariate differences in environmental features, prokaryotes and macrofauna at different longitudes and depths.

| **Water column (T, Salinity, O_2_)** | | | | |  | **Grain size** | | | | |
| --- | --- | --- | --- | --- | --- | --- | --- | --- | --- | --- |
| Source | df | MS | F | P |  | Source | df | MS | F | P |
| **Longitude** | 5 | 54.173 | 60.183 | *** |  | **Longitude** | 5 | 6.998 | 77.565 | *** |
| **Depth** | 2 | 0.598 | 0.664 | ns |  | **Depth** | 2 | 0.053 | 0.585 | ns |
| **LxD** | 10 | 2.350 | 2.611 | ns |  | **LxD** | 10 | 1.466 | 16.242 | *** |
| **Residual** | 45 | 0.900 |  |  |  | **Residual** | 36 | 0.090 |  |  |
| **Total** | 53 |  |  |  |  | **Total** | 53 |  |  |  |
|  |  |  |  |  |  |  |  |  |  |  |
| **Organic matter quantity** | | | | |  | **Organic matter quality** | | | | |
| Source | df | MS | F | P |  | Source | df | MS | F | P |
| **Longitude** | 5 | 16.737 | 65.730 | *** |  | **Longitude** | 5 | 17.969 | 16.360 | *** |
| **Depth** | 2 | 17.459 | 68.565 | *** |  | **Depth** | 2 | 2.652 | 2.415 | ns |
| **LxD** | 10 | 3.123 | 12.265 | *** |  | **LxD** | 10 | 2.431 | 2.214 | *** |
| **Residual** | 36 | 0.255 |  |  |  | **Residual** | 36 | 1.098 |  |  |
| **Total** | 53 |  |  |  |  | **Total** | 53 |  |  |  |
|  |  |  |  |  |  |  |  |  |  |  |
| **Prokaryotic stock** | | | | |  | **Macrofauna abundance** | | | | |
| Source | df | MS | F | P |  | Source | df | MS | F | P |
| **Longitude** | 5 | 16.056 | 86.695 | *** |  | **Longitude** | 5 | 7.558 | 156.790 | *** |
| **Depth** | 2 | 1.076 | 5.807 | *** |  | **Depth** | 2 | 2.773 | 57.519 | *** |
| **LxD** | 10 | 1.690 | 9.128 | *** |  | **LxD** | 10 | 0.793 | 16.452 | *** |
| **Residual** | 36 | 0.185 |  |  |  | **Residual** | 36 | 0.048 |  |  |
| **Total** | 53 |  |  |  |  | **Total** | 53 |  |  |  |
|  |  |  |  |  |  |  |  |  |  |  |
| **Macrofauna biomass** | | | | |  | **Macrofauna alpha-diversity** | | | | |
| Source | df | MS | F | P |  | Source | df | MS | F | P |
| **Longitude** | 5 | 4.066 | 25.541 | *** |  | **Longitude** | 5 | 5677.800 | 75.064 | *** |
| **Depth** | 2 | 7.317 | 45.963 | *** |  | **Depth** | 2 | 1474.300 | 19.491 | *** |
| **LxD** | 10 | 1.231 | 7.732 | *** |  | **LxD** | 10 | 532.470 | 7.040 | *** |
| **Residual** | 36 | 0.159 |  |  |  | **Residual** | 36 | 75.640 |  |  |
| **Total** | 53 |  |  |  |  | **Total** | 53 |  |  |  |
|  |  |  |  |  |  |  |  |  |  |  |
| **Macrofauna equitability index (J)** | | | | |  |  |  |  |  |  |
| Source | df | MS | F | P |  |  |  |  |  |  |
| **Longitude** | 5 | 1045.300 | 25.274 | *** |  |  |  |  |  |  |
| **Depth** | 2 | 52.593 | 1.272 | ns |  |  |  |  |  |  |
| **LxD** | 10 | 103.440 | 2.501 | ns |  |  |  |  |  |  |
| **Residual** | 45 | 41.358 |  |  |  |  |  |  |  |  |
| **Total** | 53 |  |  |  |  |  |  |  |  |  |

Indicated are: environmental features (i.e. T, salinity, grain size); organic matter quantity (i.e. CPE = chloroplastic pigments equivalent; POC flux = particulate organic carbon flux and BPC = biopolymeric organic carbon) and quality (i.e. PRT/CHO = protein to carbohydrate ratio; CPRT/BPC = protein organic carbon to biopolymeric organic carbon ratio and CCPE/BPC = phytopigment carbon to biopolymeric organic carbon ratio); prokaryotic standing stock; macrofaunal abundance and biomass, diversity and equitability.
