# Supplementary material for: Large Spatial Scale Variability in Bathyal Macrobenthos Abundance, Biomass, α- and β-Diversity along the Mediterranean Continental Margin
Source: PLoS One. 2014 Sep 16;9(9):e107261. doi: 10.1371/journal.pone.0107261 (PMC4165892; doi:10.1371/journal.pone.0107261)
Supplement: Table S3 — List of the identified macrobenthic organisms. (DOC) [file pone.0107261.s003.doc]

**Table S3.** List of the identified macrobenthic organisms.

Reported are: mean abundance values (Ind/m2±sd = standard deviation) and the corresponding trophic group (SDF = surface deposit feeder, SSDF = subsurface deposit feeder, FF = filter feeder, CNV = carnivore), at each depth along the six slope systems investigated. Names in bold are the organisms reported from all the three Mediterranean basins.
