# Supplementary material for: Large Spatial Scale Variability in Bathyal Macrobenthos Abundance, Biomass, α- and β-Diversity along the Mediterranean Continental Margin
Source: PLoS One. 2014 Sep 16;9(9):e107261. doi: 10.1371/journal.pone.0107261 (PMC4165892; doi:10.1371/journal.pone.0107261)
Supplement: Table S4 — Dissimilarity in trophic groups composition between basins and variables responsible for the estimated differences. (DOC) [file pone.0107261.s004.doc]

## **Table S4.** Dissimilarity in trophic groups composition between basins and variables responsible for the estimated differences.

|  |  |  |  | SIMPER | | ANOSIM | | |
| --- | --- | --- | --- | --- | --- | --- | --- | --- |
| **Basins** |  | **Diss. %** | **Var.** | **Contrib. %** | **Cum. %** | | **R** | **P** |
| West vs Central | | 34.85 |  |  |  | | 0.56 | *** |
|  |  |  | SSDF | 34.8 | 34.8 | |  |  |
|  |  |  | FF/SS | 29.2 | 63.9 | |  |  |
|  |  |  | CNV/SCV | 26.5 | 90.4 | |  |  |
| West vs East | | 39.31 |  |  |  | | 0.60 | ** |
|  |  |  | FF/SS | 35.3 | 35.3 | |  |  |
|  |  |  | SSDF | 28.9 | 64.3 | |  |  |
|  |  |  | CNV/SCV | 28.5 | 92.8 | |  |  |
| Central vs East | | 19.19 |  |  |  | | 0.20 | ns |
|  |  |  | CNV/SCV | 35.7 | 35.7 | |  |  |
|  |  |  | SSDF | 33.2 | 68.9 | |  |  |
|  |  |  | FF/SS | 23.1 | 92.1 | |  |  |

Reported are the results of SIMPER and ANOSIM analysis with a 90% cut of (R= sample statistic-global R; P= probability level; ***= P˂0.001; **= P˂0.01; ns= not significant). SSDF= subsurface deposit feeders; CNV/SCV= carnivores/scavengers; FF/SS= filter feeders/suspension feeders; Diss.= dissimilarity; var.= variable; Contrib.= contribution; Cum. = cumulative contribution.
