# Supplementary material for: Large Spatial Scale Variability in Bathyal Macrobenthos Abundance, Biomass, α- and β-Diversity along the Mediterranean Continental Margin
Source: PLoS One. 2014 Sep 16;9(9):e107261. doi: 10.1371/journal.pone.0107261 (PMC4165892; doi:10.1371/journal.pone.0107261)
Supplement: Table S5 — Dissimilarities in macrobenthic organisms composition between all investigated A) basins; B) slopes; C) slopes within each basin. (DOC) [file pone.0107261.s005.doc]

## **Table S5.** Dissimilarities in macrobenthic organisms composition between all investigated A) basins; B) slopes; C) slopes within each basin.

| **A)** |  |  | ANOSIM | |  | SIMPER |
| --- | --- | --- | --- | --- | --- | --- |
| **Basins** |  | **Slopes** | **R** | **P** |  | **Dissimilarity %** |
| West-Central | |  | 0.51 | ** |  | 64.65 |
| West-East | |  | 0.93 | ** |  | 81.78 |
| Central-East | |  | 0.65 | * |  | 82.17 |
| **B)** |  |  |  |  |  |  |
| West-Central | | WM1-CM1 | 0.89 | *** |  | 64.76 |
|  |  | WM1-CM2 | 0.70 | ** |  | 64.87 |
|  |  | WM2-CM1 | 0.89 | *** |  | 60.46 |
|  |  | WM2-CM2 | 0.82 | *** |  | 67.00 |
|  |  | WM3-CM1 | 0.96 | *** |  | 65.50 |
|  |  | WM3-CM2 | 0.74 | ** |  | 65.30 |
|  |  |  |  |  |  |  |
| West-East | | WM2-EM | 0.57 | ** |  | 80.90 |
|  |  | WM1-EM | 0.52 | ** |  | 80.67 |
|  |  | WM3-EM | 0.63 | ** |  | 83.76 |
|  |  |  |  |  |  |  |
| Central-East | | CM1-EM | 0.59 | ** |  | 84.69 |
|  |  | CM2-EM | 0.44 | ** |  | 79.65 |
| **C)** |  |  |  |  |  |  |
| West |  | WM1-WM2 | 0.70 | ** |  | 56.18 |
|  |  | WM1-WM3 | 0.82 | ** |  | 59.68 |
|  |  | WM2-WM3 | 0.59 | ** |  | 43.07 |
|  |  |  |  |  |  |  |
| Central |  | CM1-CM2 | 0.98 | *** |  | 71.17 |

Reported are the results of ANOSIM and SMPER analyses. SIMPER analysis run with a 90% cut of (R= sample statistic-global R; P= probability level; ***=P˂0.001; **=P˂0.01; **=P˂0.05; ns= not significant).
