# Supplementary material for: Large Spatial Scale Variability in Bathyal Macrobenthos Abundance, Biomass, α- and β-Diversity along the Mediterranean Continental Margin
Source: PLoS One. 2014 Sep 16;9(9):e107261. doi: 10.1371/journal.pone.0107261 (PMC4165892; doi:10.1371/journal.pone.0107261)
Supplement: Table S6 — Dissimilarities in the macrofaunal organisms composition between depths at all investigated slopes, from west to east basin. (DOC) [file pone.0107261.s006.doc]

## **Table S6.** Dissimilarities in the macrofaunal organisms composition between depths at all investigated slopes, from west to east basin.

|  |  |  | ANOSIM | |  | SIMPER |
| --- | --- | --- | --- | --- | --- | --- |
| **Slope** |  | **Depth (m)** | **R** | **P** | | **Dissimilarity %** |
| **WM-1** |  | 1200-1900 | 0.52 | ** | | 54.28 |
|  |  | 1200-2400 | 0.85 | ** | | 62.33 |
|  |  | 1900-2400 | 0.41 | * | | 58.76 |
| **WM-2** |  | 1200-1900 | 0.59 | ** | | 39.23 |
|  |  | 1200-2700 | 1.00 | *** | | 50.03 |
|  |  | 1900-2700 | 0.56 | ** | | 42.41 |
| **WM-3** |  | 1200-1900 | 0.52 | ** | | 44.52 |
|  |  | 1200-2400 | 0.74 | ** | | 50.60 |
|  |  | 1900-2400 | 0.06 | ns | | 43.23 |
| **CM-1** |  | 1200-1900 | 0.37 | * | | 59.70 |
|  |  | 1200-2100 | 0.85 | ** | | 68.89 |
|  |  | 1900-2100 | 0.11 | ns | | 49.18 |
| **CM-2** |  | 1200-1900 | 0.5 | * | | 55.59 |
|  |  | 1200-2700 | 1.00 | ** | | 67.00 |
|  |  | 1900-2700 | 0.5 | ns | | 58.87 |
| **EM** |  | 1200-1900 | 0.59 | ** | | 97.78 |
|  |  | 1200-2700 | 0.75 | ** | | 91.44 |
|  |  | 1900-2700 | 0.00 | ns | | 73.12 |

Reported are the results of ANOSIM and SIMPER analyses. SIMPER analysis run with a 90% cut of (R= sample statistic-global R; P= probability level; ***=P˂0.001; **=P˂0.01; **=P˂0.05; ns= not significant).
