# Supplementary material for: Large Spatial Scale Variability in Bathyal Macrobenthos Abundance, Biomass, α- and β-Diversity along the Mediterranean Continental Margin
Source: PLoS One. 2014 Sep 16;9(9):e107261. doi: 10.1371/journal.pone.0107261 (PMC4165892; doi:10.1371/journal.pone.0107261)
Supplement: Table S7 — A) Contribution of macrobenthic organisms responsible for the dissimilarity between depths. B) Contribution of macrobenthic organisms responsible for the dissimilarity between basin. (DOC) [file pone.0107261.s007.doc]

**Table S7A.** Contribution of macrobenthic organisms responsible for the dissimilarity between depths.

| **Slope** | **Depth (m)** | | **Organism** | | **Cont. %** | | |  | | **Slope** | **Depth (m)** | | **Organism** | | | **Cont. %** |
| --- | --- | --- | --- | --- | --- | --- | --- | --- | --- | --- | --- | --- | --- | --- | --- | --- |
| WM-1 | 1200-1800 | | *Golfingia sp1* | | 3.82 | | |  | | WM-2 | 1200-2700 | | Allogromiidae sp1 | | | 2.32 |
|  |  | | *Golfingia sp2* | | 3.82 | | |  | |  |  | | Ampharetidae sp1 | | | 2.32 |
|  |  | | *Nucula sp1* | | 3.82 | | |  | |  |  | | Cop. harpacticoida sp2 | | | 2.32 |
|  |  | | Eunicidae sp1 | | 2.74 | | |  | |  |  | | Fauveliopsidae sp1 | | | 2.32 |
|  |  | | *Truncorotalia sp1* | | 2.74 | | |  | |  |  | | Foraminifera sp1 | | | 2.32 |
|  |  | | Cop. harpacticoida sp1 | | 2.66 | | |  | |  |  | | *Hoeglundina elegans* | | | 2.32 |
|  |  | | *Glycera sp1* | | 2.66 | | |  | |  |  | | *Leptognathia cf. filiformis* | | | 2.32 |
|  |  | | *Leptognathia filiformis* | | 2.66 | | |  | |  |  | | Onuphiidae sp1 | | | 2.32 |
|  |  | | *Uvigerina mediterranea* | | 2.66 | | |  | |  |  | | *Uvigerina mediterranea* | | | 2.32 |
|  |  | | Ampharetidae sp1 | | 2.35 | | |  | |  |  | | Ilyarachnidae sp1 | | | 1.62 |
|  |  | | Paraonidae sp2 | | 2.35 | | |  | |  |  | | Paraonidae sp1 | | | 1.62 |
|  |  | | Onuphiidae sp1 | | 2.32 | | |  | |  |  | | Desmosomatidae sp2 | | | 1.59 |
|  |  | | *Paranarthrura sp1* | | 2.32 | | |  | |  |  | | *Golfingia sp2* | | | 1.59 |
|  |  | | Siphonodentaliidae sp1 | | 2.32 | | |  | |  |  | | Ischnomesidae sp1 | | | 1.52 |
|  |  | | Oligochaeta sp2 | | 2.08 | | |  | |  |  | | Pilargidae sp1 | | | 1.52 |
|  |  | | Paraonidae sp1 | | 2.08 | | |  | |  |  | | *Golfingia sp1* | | | 1.52 |
|  |  | | Oligochaeta sp1 | | 2.06 | | |  | |  |  | | Cuspidariidae sp1 | | | 1.51 |
|  |  | | Syllidae sp1 | | 2.03 | | |  | |  |  | | Arenicolidae sp1 | | | 1.51 |
|  |  | | Eusiridae sp1 | | 1.79 | | |  | |  |  | | *Anthelura sp1* | | | 1.51 |
|  |  | | Ischnomesidae sp1 | | 1.76 | | |  | |  |  | | Terebellidae sp1 | | | 1.51 |
| WM-1 | 1200-2400 | | *Cuspidaria sp1* | | 3.86 | | |  | | WM-2 | 1900-2700 | | Ampharetidae sp1 | | | 3.32 |
|  |  | | *Glycera sp1* | | 3.86 | | |  | |  |  | | *Hoeglundina elegans* | | | 3.32 |
|  |  | | *Golfingia sp1* | | 3.86 | | |  | |  |  | | *Limopsis sp1* | | | 3.32 |
|  |  | | *Nucula sp1* | | 3.86 | | |  | |  |  | | Anthuriidae sp1 | | | 2.3 |
|  |  | | *Uvigerina mediterranea* | | 3.86 | | |  | |  |  | | Oweniidae sp1 | | | 2.3 |
|  |  | | Eunicidae sp1 | | 2.8 | | |  | |  |  | | *Golfingia sp2* | | | 2.3 |
|  |  | | *Truncorotalia sp1* | | 2.8 | | |  | |  |  | | *Uvigerina mediterranea* | | | 2.28 |
|  |  | | *Pseudotrachya hystrix* | | 2.61 | | |  | |  |  | | Allogromiidae sp1 | | | 2.2 |
|  |  | | Capitellidae sp1 | | 2.6 | | |  | |  |  | | Bryozoa sp3 | | | 2.2 |
|  |  | | *Bathyarca sp1* | | 2.58 | | |  | |  |  | | Terebellidae sp1 | | | 2.2 |
|  |  | | *Hoeoglundina elegans* | | 2.58 | | |  | |  |  | | *Yoldiella sp1* | | | 2.2 |
|  |  | | Heterospionidae sp1 | | 2.58 | | |  | |  |  | | Bryozoa sp1 | | | 2.18 |
|  |  | | *Limopsis sp1* | | 2.58 | | |  | |  |  | | Ischnomesidae sp1 | | | 2.18 |
|  |  | | *Sclerochilus sp1* | | 2.58 | | |  | |  |  | | *Golfingia sp1* | | | 2.18 |
|  |  | | Cop. harpacticoida sp1 | | 2.52 | | |  | |  |  | | Cop. harpacticoida sp2 | | | 2.17 |
|  |  | | Onuphiidae sp1 | | 2.31 | | |  | |  |  | | Ostracoda sp1 | | | 2.17 |
|  |  | | *Paranarthrura sp1* | | 2.31 | | |  | |  |  | | Desmosomatidae sp2 | | | 1.87 |
|  |  | | Siphonodentaliidae sp1 | | 2.06 | | |  | |  |  | | *Ammolagena clavata* | | | 1.85 |
|  |  | | Oligochaeta sp1 | | 1.8 | | |  | |  |  | | *Leptognathia unguicillata* | | | 1.85 |
|  |  | | Syllidae sp1 | | 1.8 | | |  | |  |  | | Poecilochaetidae sp1 | | | 1.85 |
| WM-2 | 1200-1900 | | Fauveliopsidae sp1 | | 2.62 | | |  | | WM-3 | 1200-1900 | | Fauveliopsidae sp1 | | | 2.58 |
|  |  | | *Leptognathia cf. filiformis* | | 2.62 | | |  | |  |  | | Paraonidae sp1 | | | 2.58 |
|  |  | | *Limopsis sp1* | | 2.62 | | |  | |  |  | | *Uvigerina mediterranea* | | | 2.58 |
|  |  | | Onuphiidae sp1 | | 2.62 | | |  | |  |  | | Fauveliopsidae sp2 | | | 1.75 |
|  |  | | Pilargidae sp1 | | 2.62 | | |  | |  |  | | Ischnomesidae sp1 | | | 1.75 |
|  |  | | *Pseodotanais macrocheles* | | 1.83 | | |  | |  |  | | Lyssianassidae sp1 | | | 1.75 |
|  |  | | *Ammolagena clavata* | | 1.78 | | |  | |  |  | | Nemertea sp1 | | | 1.75 |
|  |  | | Foraminifera sp1 | | 1.78 | | |  | |  |  | | *Pseudotrachya hystrix* | | | 1.75 |
|  |  | | *Leptognathia unguicillata* | | 1.78 | | |  | |  |  | | *Campylaspis sp1* | | | 1.7 |
|  |  | | Bryozoa sp3 | | 1.74 | | |  | |  |  | | *Collettea cylindrata* | | | 1.7 |
|  |  | | Ostracoda sp1 | | 1.72 | | |  | |  |  | | Heterospionidae sp2 | | | 1.7 |
|  |  | | Dentaliida sp1 | | 1.72 | | |  | |  |  | | Phoxocephalidae sp1 | | | 1.7 |
|  |  | | *Anthelura sp1* | | 1.72 | | |  | |  |  | | Serpulidae sp1 | | | 1.7 |
|  |  | | *Arca sp1* | | 1.7 | | |  | |  |  | | *Yoldiella sp1* | | | 1.7 |
|  |  | | *Collettea cylindrata* | | 1.7 | | |  | |  |  | | Cossuridae sp1 | | | 1.7 |
|  |  | | *Glycera sp2* | | 1.7 | | |  | |  |  | | *Glycera sp1* | | | 1.7 |
|  |  | | *Harpinia truncata* | | 1.7 | | |  | |  |  | | *Sclerochilus sp1* | | | 1.7 |
|  |  | | *Sclerochilus sp1* | | 1.7 | | |  | |  |  | | Iospilidae sp1 | | | 1.67 |
|  |  | | Ilyarachnidae sp1 | | 1.5 | | |  | |  |  | | *Limopsis sp1* | | | 1.67 |
|  |  | | Paraonidae sp1 | | 1.47 | | |  | |  |  | | Sabellidae sp1 | | | 1.67 |
| **Slope** | **Depth (m)** | **Organism** | | **Cont. %** | |  | **Slope** | | **Depth (m)** | | | **Organism** | | **Cont. %** |  | |
| WM-3 | 1200-2400 | Ampharetidae sp1 | | 2.55 | |  | CM-2 | | 1200-2700 | | | Aphroditidae sp1 | | 5.08 |  | |
|  |  | Fauveliopsidae sp1 | | 2.55 | |  |  | |  | | | *Golfingia sp2* | | 5.08 |  | |
|  |  | Onuphiidae sp1 | | 2.55 | |  |  | |  | | | Heterospionidae sp1 | | 5.08 |  | |
|  |  | *Pseudotrachya hystrix* | | 2.55 | |  |  | |  | | | *Pseudotrachya hystrix* | | 5.08 |  | |
|  |  | *Uvigerina mediterranea* | | 2.55 | |  |  | |  | | | Syllidae sp1 | | 5.08 |  | |
|  |  | Rissoidea sp1 | | 1.77 | |  |  | |  | | | Terebellidae sp1 | | 5.08 |  | |
|  |  | Spionidae sp2 | | 1.77 | |  |  | |  | | | Cirratulidae sp2 | | 3.63 |  | |
|  |  | Cossuridae sp1 | | 1.72 | |  |  | |  | | | *Golfingia sp1* | | 3.63 |  | |
|  |  | *Sclerochilus sp1* | | 1.72 | |  |  | |  | | | Capitellidae sp1 | | 3.2 |  | |
|  |  | Terebellidae sp1 | | 1.72 | |  |  | |  | | | Cuspidariidae sp1 | | 3.2 |  | |
|  |  | Ischnomesidae sp1 | | 1.7 | |  |  | |  | | | *Limopsis sp1* | | 3.2 |  | |
|  |  | *Physcosoma sp1* | | 1.7 | |  |  | |  | | | Oligochaeta sp1 | | 3.2 |  | |
|  |  | Syllidae sp1 | | 1.7 | |  |  | |  | | | *Truncorotalia sp1* | | 3.2 |  | |
|  |  | *Campylaspis sp1* | | 1.68 | |  |  | |  | | | Paraonidae sp1 | | 2.61 |  | |
|  |  | *Desmosoma sp1* | | 1.68 | |  |  | |  | | | Cirratulidae sp1 | | 2.58 |  | |
|  |  | Eunicidae sp1 | | 1.68 | |  |  | |  | | | *Ammolagena clavata* | | 2.52 |  | |
|  |  | Heterospionidae sp2 | | 1.68 | |  |  | |  | | | Bryozoa sp1 | | 2.5 |  | |
|  |  | Phoxocephalidae sp1 | | 1.68 | |  |  | |  | | | Cop. harpacticoida sp1 | | 2.41 |  | |
|  |  | *Yoldiella sp1* | | 1.68 | |  |  | |  | | | *Glycera sp1* | | 2.41 |  | |
|  |  | *Leptognathia unguicillata* | | 1.67 | |  |  | |  | | | Hesionidae sp1 | | 2.41 |  | |
| CM-1 | 1200-2100 | Heterospionidae sp1 | | 3.81 | |  | EM | | 1200-1900 | | | *Golfingia sp1* | | 11.09 |  | |
|  |  | Heterospionidae sp2 | | 3.81 | |  |  | |  | | | Heterospionidae sp1 | | 7.89 |  | |
|  |  | Ischnomesidae sp1 | | 3.81 | |  |  | |  | | | Capitellidae sp1 | | 7.54 |  | |
|  |  | Nemertea sp1 | | 3.81 | |  |  | |  | | | Oweniidae sp1 | | 7.54 |  | |
|  |  | Oligochaeta sp1 | | 3.81 | |  |  | |  | | | Sipuncula sp1 | | 7.54 |  | |
|  |  | Sipuncula sp1 | | 3.81 | |  |  | |  | | | *Stephanoscyphus sp1* | | 7.54 |  | |
|  |  | Cop. harpacticoida sp2 | | 2.73 | |  |  | |  | | | Serpulidae sp1 | | 4.91 |  | |
|  |  | Eusiridae sp1 | | 2.72 | |  |  | |  | | | Syllidae sp1 | | 3.98 |  | |
|  |  | Cop. harpacticoida sp3 | | 2.51 | |  |  | |  | | | Ampharetidae sp1 | | 3.56 |  | |
|  |  | Chaetopteridae sp1 | | 2.39 | |  |  | |  | | | Aphroditidae sp1 | | 3.56 |  | |
|  |  | Syllidae sp1 | | 2.17 | |  |  | |  | | | *Arca sp1* | | 3.56 |  | |
|  |  | Arabellidae sp1 | | 2.13 | |  |  | |  | | | *Dyastiloides sp1* | | 3.56 |  | |
|  |  | Cossuridae sp1 | | 2.13 | |  | EM | | 1200-2700 | | | *Golfingia sp1* | | 10.69 |  | |
|  |  | *Glycera sp1* | | 2.13 | |  |  | |  | | | Oweniidae sp1 | | 7.25 |  | |
|  |  | Oligochaeta sp2 | | 2.13 | |  |  | |  | | | Sipuncula sp1 | | 7.25 |  | |
|  |  | *Kelliella sp1* | | 2.1 | |  |  | |  | | | Heterospionidae sp1 | | 6.91 |  | |
|  |  | Macrostylidae sp1 | | 2 | |  |  | |  | | | Capitellidae sp1 | | 6.14 |  | |
|  |  | Cop. harpacticoida sp1 | | 1.98 | |  |  | |  | | | *Stephanoscyphus sp1* | | 5.9 |  | |
|  |  | Spionidae sp2 | | 1.96 | |  |  | |  | | | Serpulidae sp1 | | 3.81 |  | |
|  |  | Macrostylidae sp2 | | 1.68 | |  |  | |  | | | Syllidae sp1 | | 3.81 |  | |
|  |  |  | |  | |  |  | |  | | | Cop. harpacticoida sp1 | | 3.78 |  | |
|  |  |  | |  | |  |  | |  | | | *Glycera sp1* | | 3.78 |  | |
|  |  |  | |  | |  |  | |  | | | Ampharetidae sp1 | | 3.44 |  | |
|  |  |  | |  | |  |  | |  | | | Aphroditidae sp1 | | 3.44 |  | |
|  |  |  | |  | |  |  | |  | | | *Arca sp1* | | 3.44 |  | |
|  |  |  | |  | |  |  | |  | | | *Dyastiloides sp1* | | 3.44 |  | |

Reported are: percentage contribution to the dissimilarity (Cont. %) of the first twenty organisms, where present, based on SIMPER analysis between depths along the same slope.

**Table S7B.** Contribution of macrobenthic organisms responsible for the dissimilarity between basin.

| **Basin** | **Organism** | **Cont. %** |  | **Basin** | **Organism** | **Cont. %** |
| --- | --- | --- | --- | --- | --- | --- |
| West-Central | Maldanidae sp1 | 1.85 |  | West-East | Cirratulidae sp1 | 1.95 |
|  | *Truncorotalia sp1* | 1.56 |  |  | Oligochaeta sp1 | 1.95 |
|  | Fauveliopsidae sp1 | 1.54 |  |  | Oligochaeta sp2 | 1.95 |
|  | Capitellidae sp2 | 1.36 |  |  | Spionidae sp1 | 1.95 |
|  | *Pseodotanais macrocheles* | 1.34 |  |  | *Truncorotalia sp1* | 1.95 |
|  | Capitellidae sp1 | 1.19 |  |  | Fauveliopsidae sp1 | 1.66 |
|  | Ischnomesidae sp1 | 1.16 |  |  | Capitellidae sp2 | 1.62 |
|  | Hydroida sp1 | 1.15 |  |  | Ischnomesidae sp1 | 1.62 |
|  | Serpulidae sp1 | 1.14 |  |  | Paraonidae sp1 | 1.54 |
|  | *Kelliella sp1* | 1.12 |  |  | *Pseudotrachya hystrix* | 1.46 |
|  | Syllidae sp2 | 1.12 |  |  | Maldanidae sp1 | 1.38 |
|  | Poecilochaetidae sp1 | 1.09 |  |  | *Metasphaerolaimus sp1* | 1.38 |
|  | Oligochaeta sp1 | 1.05 |  |  | Syllidae sp1 | 1.38 |
|  | Oligochaeta sp2 | 1.05 |  |  | *Pseodotanais macrocheles* | 1.37 |
|  | *Golfingia sp2* | 1.04 |  |  | Cirratulidae sp2 | 1.35 |
|  | Cossuridae sp1 | 1.04 |  |  | Cop. harpacticoida sp1 | 1.28 |
|  | *Limopsis sp1* | 1.02 |  |  | Syllidae sp2 | 1.24 |
|  | Fauveliopsidae sp2 | 1.01 |  |  | Cossuridae sp1 | 1.23 |
|  | Orbinidae sp1 | 1.00 |  |  | Anthuriidae sp1 | 1.19 |
|  | Ostracoda sp1 | 0.97 |  |  | *Glycera sp1* | 1.18 |
|  |  |  |  |  |  |  |
|  |  |  |  |  |  |  |
| **Basin** | **Organism** | **Cont. %** |  |  |  |  |
| Central-East | Paraonidae sp1 | 3.49 |  |  |  |  |
|  | Cirratulidae sp1 | 3.09 |  |  |  |  |
|  | Spionidae sp1 | 2.72 |  |  |  |  |
|  | *Metasphaerolaimus sp1* | 2.58 |  |  |  |  |
|  | Serpulidae sp1 | 2.28 |  |  |  |  |
|  | Spionidae sp2 | 2.26 |  |  |  |  |
|  | *Ammolagena clavata* | 2.2 |  |  |  |  |
|  | *Pseudotrachya hystrix* | 2.18 |  |  |  |  |
|  | Cop. harpacticoida sp1 | 2.13 |  |  |  |  |
|  | Syllidae sp1 | 2.07 |  |  |  |  |
|  | *Kelliella sp1* | 2.07 |  |  |  |  |
|  | *Glycera sp1* | 1.99 |  |  |  |  |
|  | Capitellidae sp1 | 1.92 |  |  |  |  |
|  | Ampharetidae sp1 | 1.82 |  |  |  |  |
|  | Eusiridae sp1 | 1.78 |  |  |  |  |
|  | Cirratulidae sp2 | 1.76 |  |  |  |  |
|  | *Stephanoscyphus sp1* | 1.69 |  |  |  |  |
|  | *Nucula sp1* | 1.68 |  |  |  |  |
|  | Heterospionidae sp1 | 1.67 |  |  |  |  |
|  |  |  |  |  |  |  |

Reported are: percentage contribution to the dissimilarity (Cont. %) of the first twenty organisms, where present, based on SIMPER analysis between basins.
