# Supplementary material for: Large Spatial Scale Variability in Bathyal Macrobenthos Abundance, Biomass, α- and β-Diversity along the Mediterranean Continental Margin
Source: PLoS One. 2014 Sep 16;9(9):e107261. doi: 10.1371/journal.pone.0107261 (PMC4165892; doi:10.1371/journal.pone.0107261)
Supplement: Table S8 — Results of the multivariate multiple regression analysis carried out on the macrofaunal descriptors. (DOC) [file pone.0107261.s008.doc]

***Table S8****. Results of the multivariate multiple regression analysis carried out on the macrofaunal descriptors.*

| **Dependent Var.** | **Explan. Var.** | | **F** | **P** | **% Var.** | **% Cum.** |
| --- | --- | --- | --- | --- | --- | --- |
| Abundance | BPC | 18.786 | | *** | 26.5 | 26.5 |
|  | Grain size | 10.548 | | ** | 12.6 | 39.1 |
|  | TPN | 5.667 | | * | 6.1 | 45.2 |
|  | PRT/CHO | 4.957 | | * | 2.8 | 48.0 |
| Biomass | BPC | 14.914 | | ** | 22.3 | 22.3 |
|  | Grain size | 11.861 | | ** | 14.7 | 37 |
| SR. ES(n) | BPC | 5.616 | | ** | 9.8 | 9.8 |
|  | POC flux | 5.220 | | ** | 8.5 | 18.3 |
|  | Grain size | 5.994 | | ** | 7.8 | 26.1 |
|  | CPRT | 4.201 | | * | 6.1 | 32.2 |
|  | TPN |  | | * | 5.4 | 37.6 |
| n° Taxa | CPRT | 15.281 | | *** | 22.7 | 22.7 |
|  | Grain size | 7.588 | | ** | 9.3 | 32.0 |
|  | BPC | 4.639 | | * | 6.5 | 38.5 |
|  | POC flux | 4.487 | | * | 5.2 | 43.7 |
|  | CCPE | 3.906 | | * | 4.3 | 48.0 |
| SDF | PRT/CHO | 12.530 | | *** | 19.4 | 19.4 |
|  | CCPE | 4.025 | | * | 6.1 | 25.5 |
| SSDF | TPN | 11.422 | | ** | 18.3 | 18.3 |
| CNV/SCV | TPB | 9.934 | | ** | 14.6 | 14.6 |
| FF/SS | variables | all ns | |  |  |  |

Reported are: abundance, biomass, species richness (SR) and expected species number (ES(n)), number of taxa, trophic community structure (% of SDF = surface deposit feeder, SSDF = subsurface deposit feeder, FF = filter feeder, CNV = carnivore) of all investigated areas. Explan. Var. = explanatory variable; % Var = percentage of explained variance ( F= F-statistic; P= probability level; *** =P˂0.001; **=P˂0.01; *=P˂0.05; ns= not significant). Reported are only significant environmental variables for each of the macrofauna descriptors (Grain= grain size).
