# Supplementary material for: Large Spatial Scale Variability in Bathyal Macrobenthos Abundance, Biomass, α- and β-Diversity along the Mediterranean Continental Margin
Source: PLoS One. 2014 Sep 16;9(9):e107261. doi: 10.1371/journal.pone.0107261 (PMC4165892; doi:10.1371/journal.pone.0107261)
Supplement: Table S9 — Results of the multivariate multiple regression analysis carried out separately in the western, central and eastern basins on the macrofaunal descriptors. (DOC) [file pone.0107261.s009.doc]

**Table S9***.* Results of the multivariate multiple regression analysis carried out separately in the western, central and eastern basins on the macrofaunal descriptors.

| **Basin** | **Dependent var.** | **Explan. var.** | **F** | **P** | **% var.** | **% cum.** |
| --- | --- | --- | --- | --- | --- | --- |
| West | Abundance | CPRT | 34.472 | *** | 58.0 | 58.0 |
| (N= 27) |  | TPN | 5.196 | * | 7.5 | 65.5 |
|  | Biomass | CPRT | 34.708 | *** | 58.1 | 58.1 |
|  |  | PRT/CHO | 4.878 | * | 7.1 | 65.2 |
|  | SR. ES(n) | PRT/CHO | 7.269 | ** | 22.5 | 22.5 |
|  |  | CPRT | 6.263 | * | 16.1 | 38.6 |
|  | n° Taxa | PRT/CHO | 19.855 | *** | 44.3 | 44.3 |
|  |  | Grain size | 7.038 | * | 10.5 | 54.8 |
|  |  | TPN | 5.943 | * | 11.1 | 65.9 |
|  | SDF | CCPE | 4.059 | * | 14.0 | 14.0 |
|  | SSDF | all | ns |  |  |  |
|  | CNV/SCV | TPN | 4.556 | * | 15.4 | 15.4 |
|  | FF/SS | BPC | 4.404 | * | 15.0 | 15.0 |
|  |  |  |  |  |  |  |
| Central | Abundance | CPRT | 18.690 | ** | 53.9 | 53.9 |
| (N= 18) | Biomass | BPC | 31.623 | *** | 43.2 | 43.2 |
|  |  | PRT/CHO | 4.725 | * | 22.8 | 66.0 |
|  | SR. ES(n) | BPC | 12.077 | ** | 43.1 | 43.1 |
|  | n° Taxa | BPC | 8.438 | ** | 34.5 | 34.5 |
|  | SDF | TPN | 28.624 | *** | 64.1 | 64.1 |
|  |  | BPC | 6.979 | ** | 9.3 | 73.4 |
|  | SSDF | POC flux | 9.947 | ** | 38.4 | 38.4 |
|  |  | PRT/CHO | 13.570 | ** | 29.3 | 67.7 |
|  |  | BPC | 5.811 | ** | 9.5 | 77.2 |
|  | FF/SS | TPN | 4.425 | * | 21.7 | 21.7 |
|  |  | Grain size | 3.699 | * | 15.5 | 37.2 |
|  | CNV/SCV | TPN | 6.797 | ** | 29.8 | 29.8 |
|  |  | BPC | 3.145 | * | 11.4 | 41.2 |
|  |  |  |  |  |  |  |
| East | Abundance | BPC | 29.701 | ** | 80.9 | 80.9 |
| (N= 9) | Biomass | BPC | 16.456 | ** | 70.2 | 70.2 |
|  | SR. ES(n) | BPC | 13.075 | ** | 40.5 | 40.5 |
|  |  | Grain size | 4.840 | * | 40.9 | 81.4 |
|  | n° Taxa | BPC | 7.693 | * | 52.4 | 52.4 |
|  |  | Grain size | 4.202 | * | 19.6 | 72.0 |
|  |  | POC flux | 5.128 | * | 14.2 | 86.2 |
|  | SDF | all | ns |  |  |  |
|  | SSDF | Grain size | 3.906 | * | 22.2 | 22.2 |
|  | FF/SS | POC flux | 31.565 | ** | 81.9 | 81.9 |
|  |  | CCPE | 8.278 | * | 6.0 | 87.9 |
|  |  | BPC | 9.262 | * | 2.2 | 90.1 |
|  | CNV/SCV | BPC | 5.964 | * | 46.0 | 46.0 |
|  |  | Grain size | 4.779 | * | 23.9 | 69.9 |

Reported are: abundance, biomass, diversity (including SR, ES(n) and number of taxa), trophic community structure (% of SDF = surface deposit feeder, SSDF = subsurface deposit feeder, FF = filter feeder, CNV = carnivore). Explan. Var. = explanatory variable; % Var = percentage of explained variance ( F= F-statistic; P= probability level; *** =P˂0.001; **=P˂0.01; *=P˂0.05; ns= not significant); % Cum. = total cumulative percentage. Reported are only significant environmental variables for each of the macrofauna descriptors (grain= grain size). N= number of dependant variable values on which the analysis has been run.
